# Taxon-Function Decoupling as an Adaptive Signature of Lake Microbial Metacommunities Under a Chronic Polymetallic Pollution Gradient

**DOI:** 10.3389/fmicb.2018.00869

**Published:** 2018-05-03

**Authors:** Bachar Cheaib, Malo Le Boulch, Pierre-Luc Mercier, Nicolas Derome

**Affiliations:** ^1^Institut de Biologie Intégrative et des Systèmes, Université Laval, Quebec, QC, Canada; ^2^GenPhySE, Institut National de la Recherche Agronomique, Université de Toulouse, INPT, ENVT, Castanet-Tolosan, France

**Keywords:** function, taxon, decoupling, polymetallic gradient, Cadmium, evolution, adaptation, resistance

## Abstract

Adaptation of microbial communities to anthropogenic stressors can lead to reductions in microbial diversity and disequilibrium of ecosystem services. Such adaptation can change the molecular signatures of communities with differences in taxonomic and functional composition. Understanding the relationship between taxonomic and functional variation remains a critical issue in microbial ecology. Here, we assessed the taxonomic and functional diversity of a lake metacommunity system along a polymetallic pollution gradient caused by 60 years of chronic exposure to acid mine drainage (AMD). Our results highlight three adaptive signatures. First, a signature of taxon—function decoupling was detected in the microbial communities of moderately and highly polluted lakes. Second, parallel shifts in taxonomic composition occurred between polluted and unpolluted lakes. Third, variation in the abundance of functional modules suggested a gradual deterioration of ecosystem services (i.e., photosynthesis) and secondary metabolism in highly polluted lakes. Overall, changes in the abundance of taxa, function, and more importantly the polymetallic resistance genes such as *copA, copB, czcA, cadR, cCusA*, were correlated with trace metal content (mainly Cadmium) and acidity. Our findings highlight the impact of polymetallic pollution gradient at the lowest trophic levels.

## Introduction

Micro-organisms represent a significant portion of global biodiversity and are the engine driving Earth's biogeochemical cycles and primary production (Falkowski et al., 2008; Green et al., 2008). Ecosystem services provided by microbes ensure optimal environmental conditions for all multicellular life forms (Robinson et al., 2010). For decades, the implications of taxon-function relationships in microbial communities have been debated by researchers (Doolittle and Zhaxybayeva, 2009; Bissett et al., 2013; Martiny et al., 2013; Louca et al., 2016b; Morrissey et al., 2016). On one hand, researchers showed that even very closely related taxa exhibited contrasting metabolic and ecological functions (e.g., distinct growth rates and metabolic substrate utilization profiles), indicating a gap between taxon phylogeny and the functional repertoires of some bacterial genera (Jaspers and Overmann, 2004; Maharjan et al., 2006; Doolittle and Zhaxybayeva, 2009). These studies employed molecular taxonomic profiling, either by sequencing SSU (small subunit ribosomal ribonucleic acid) 16S rRNA (Jaspers and Overmann, 2004; Doolittle and Zhaxybayeva, 2009) or specific housekeeping genes (Maharjan et al., 2006). On the other hand, studies focused on microbial molecular evolution and ecology reported a significant relationship between phylogenetic groups or taxonomic composition at different hierarchical levels (i.e., Phylum and Class) with ecological and functional traits (Webb et al., 2002; Martiny et al., 2006, 2013; Ward et al., 2006; Gupta and Lorenzini, 2007; Allison and Martiny, 2008; Philippot et al., 2010; Gravel et al., 2011). The majority of these genomic studies have been limited to correlating traits with taxa abundance variation. Additional evidence at the community level is needed to predict the interplay of evolutionary processes [horizontal gene transfer (HGT), gene loss, selective pressure] and ecological processes (spatial dispersal limits, biotic interactions, neutral biogeography) drive metacommunity composition and functional repertoires in complex ecological contexts.

With advances in sequencing technologies, metagenomic approaches have the potential to advance our understanding of both the taxonomic and functional composition of complex microbial communities. In this respect, metagenomic studies have revealed significant coupling between taxonomic composition or phylogenetic lineages and ecological traits (Bouvier and del Giorgio, 2002; Philippot et al., 2010) or functional gene repertoires (Debroas et al., 2009; Goldfarb et al., 2011; Muegge et al., 2011; Bryant et al., 2012; Fierer et al., 2012b; Langille et al., 2013; Martiny et al., 2013; Forsberg et al., 2014; Mayali et al., 2014; Vanwonterghem et al., 2014; Morrissey et al., 2016; Larkin and Martiny, 2017). For example, in natural lake communities, associations are reported between taxon abundance and function (Debroas et al., 2009), and in soil communities from multiple environments, with chemical substrate variation (Goldfarb et al., 2011) and functional attributes (Fierer et al., 2012b). Most of these studies have been conducted in relatively unperturbed environments, and on microbial communities facing moderate to low selective pressure.

Other microbial community studies, mostly based on 16S rRNA gene analysis, and rarely complemented by whole metagenome shotgun sequencing, revealed either partial or marked decoupling between taxonomic composition and ecological traits (Lima-Mendez et al., 2015) or functional gene repertoires. Patterns of complete to partial decoupling are often found in natural environmental conditions (Hooper et al., 2008, 2009; Burke et al., 2011; Raes et al., 2011; Smillie et al., 2011; Barberán et al., 2012; Louca et al., 2016b). This taxon-function decoupling has rarely been discussed in extreme environments such as acid mine drainages (AMD) (Kuang et al., 2016). These findings highlight the need to further investigate environments where initial conditions have been perturbed by xenobiotic factors (Bowen et al., 2011).

The occurrence of taxon-function decoupling has been reported in other metagenomic studies as functional redundancy between phylogenetically distant taxa (Green et al., 2008; Burke et al., 2011; Stokes and Gillings, 2011) and divergent microenvironments (Hooper et al., 2008, 2009). To summarize, taxonomic and functional features could be useful in assessing adaptive response of microbial metacommunities in disturbed ecosystems. One study, to our knowledge, has focused on the outcome of microbial taxon-function relationships under selection gradients, indicating possible linkages between the structure and functioning of soil microbial communities (Fierer et al., 2012a). Thus, it remains uncertain whether taxon-function decoupling is an adaptive response to a gradual selective pressure. Xenobiotic stressors like antibiotics, chemical and metallic pollutants erode microbial biodiversity (Parnell et al., 2009), which is predicted to impair or erode ecosystem services (Sandifer and Sutton-Grier, 2014). Therefore, the characterization of taxon-function decoupling patterns will enhance our understanding of the robustness of microbial functional networks that ensure key ecosystem services. Here, the complex connections of microbial biodiversity and ecosystem services (Miki et al., 2014) were addressed at the molecular level by comparing variation in the taxonomic composition and molecular functions of microbial communities.

We hypothesized that a stress gradient, specifically a polymetallic pollution gradient over a relatively long evolutionary time scale in terms of bacterial generation time, would result in adaptive signatures in taxonomic composition and functional repertoires. Specifically, we predicted that stress gradients would gradually induce selection for microbial metacommunities with functional repertoires and a taxonomic composition capable of thriving in this harsh environment. To test our hypothesis, we targeted lakes polluted by a polymetallic gradient of acid mine waters. Heavy metals can originate either from natural sources such as volcanic activity or anthropogenically by mines tailings, an important source of AMD. Acidity gradients recorded in lake waters surrounded by natural volcanic activity (e.g., Indonesian crater lake Kawah Ijen, Argentinian volcanic lake in Patagonia), have significant effects on the microbial community composition and biodiversity (Wendt-Potthoff and Koschorreck, 2002; Löhr et al., 2006). AMD is created by the exposure of sulphidic minerals to air and water forming soluble sulfates (Almeida et al., 2008). Ferrous minerals become oxidized in contact with water producing ferric ions and H_2_ (Johnson and Hallberg, 2003; Edwards and Bazylinski, 2008). Leached ions into streams generate acidic water by lowering the pH (<3). Consequently, other metal ions such as Zn, Hg, Ni, Cr, Cd, Cu, Mn, Al, As, and Pb appear in AMD waters at high concentrations. There are limited descriptions of microbial diversity in AMD in the literature, especially in impacted environments with high zinc and cadmium concentrations (Almeida et al., 2008). In AMD polluted surface water, Almeida et al. (2008) showed that bacterial diversity in Sepetiba Bay, Brazil, which is much higher than archaeal diversity, was dominated by *Proteobacteria, Actinobacteria, Cyanobacteria* and had a high abundance of unclassified bacteria (unknown strains). Similar composition (dominance of Proteobacteria) was observed over 59 microbial communities from physically and geochemically diverse AMD sites across Southeast China (Kuang et al., 2013). Kuang et al. (2013) revealed that acidity gradient is a major factor explaining community differences between AMD communities regardless of the long-distance isolation and the distinct substrate types. Likewise, the investigation of the microbial diversity of an extremely acidic, metal-rich water lake (Lake Robule, Bor, Serbia) revealed low diversity dominated by *Proteobacteria* strains (Stankovic et al., 2014). Similar community composition was observed in bacterioplankton communities exposed to cadmium in coastal water microcosms (Wang et al., 2015). Similar to surface waters, Hemme et al. (2010, 2016) highlighted that chronic exposure to high concentrations of heavy metals (~50 years) in groundwater caused a massive decrease in biodiversity, characterized by a high abundance of *Proteobacteria*, as well as a significant loss in allelic and metabolic diversity. More importantly, Hemme et al. (2016) pointed to the importance of HGT during the evolution of groundwater microbial communities in response to heavy metal exposure. However, very few studies were carried out on water polluted across a polymetallic gradient (Kuang et al., 2013; Desoeuvre et al., 2016). One of those studies reported the impact of an extreme poly-metallic gradient (including arsenic) on the diversity and distribution of arsenic-related genes in river waters (Desoeuvre et al., 2016). Other studies on AMD polluted freshwater sediments (Sánchez-Andrea et al., 2011; Jackson et al., 2015; Jie et al., 2016; Ni et al., 2016) showed the dominance of *Proteobacteria* in microbial communities as well as community specialization. In lake sediments exposed to AMD gradients, the effects of different metals on specific microbes and microbial activities were correlated with their respective chemical properties. All these studies used 16S rRNA gene analysis, except for one, which used deep coverage data from shotgun metagenome sequencing (Hemme et al., 2016).

In our study, we used a shotgun metagenomic sequencing approach to characterize the functional and taxonomic diversity of bacterioplankton from five lakes within a catchment that was historically exposed to a polymetallic contamination gradient (PCG) for over 60 years. As the PCG was previously correlated with taxon abundance variation (Laplante and Derome, 2011; Laplante et al., 2013), taxon-function decoupling was expected to occur in the most polluted lakes and be absent in less polluted or unpolluted lakes. Our first objective was to assess the taxonomic and functional signatures of bacterioplankton adaptation to PCG. Secondly, we aimed to provide insight into the interplay of biodiversity and ecosystem services under a stress gradient by analyzing taxon-function variation.

## Materials and methods

### Lake characteristics and locations

Over the last 60 years, the Rouyn-Noranda (Western Quebec, Canada) mining sites have dumped AMD with heavy polymetallic traces (Laplante and Derome, 2011) into surrounding lakes. We targeted five lakes in this area (Supplementary Figure S1). Among them, three have common surface water interconnected along the same hydrologic basin: Arnoux Lake (LAR-hc; highly polluted), Arnoux Bay (BAR-mc; medium levels of pollution), and Dasserat Lake (DAS-lc; the least polluted). The water polluted by AMD spreads from Arnoux to Dasserat Lake generating a polymetallic gradient over 20 km. Around 30 km to the south side of this natural system of connected lakes, Opasatica Lake (OPA-nc), which is a landlocked unpolluted site, was sampled and considered as an unpolluted negative control, and ca. 40 km to the north side, Turcotte Lake (TUR-hc), another landlocked site was selected as a highly polluted lake. Longitude and latitude coordinates are given in Supplementary File S1. This Western Quebec lake system is 425 km northwest of Ottawa, Ontario. The abandoned mine site is a source of tailings and eroded mine waste into the Arnoux River, which drains west to Arnoux Lake, Arnoux Bay, and then Dasserat Lake. These lakes are irregular in shape and the bathymetry reflects the relief of the underlying bedrock. The immediate surrounding area consists of hilly terrain, volcanic rocks, ultramafic rocks, mafic intrusions, granitic rocks, and early and middle Precambrian sediments (Alpay, 2016).

### Metallic and chemical gradient surveys

pH and Polymetallic concentration (Al, Cd, Cu, Fe, Mn, Pb, Zn) in the studied lakes was measured in June 2010, a year prior to the present study, using ICP VISTA Varian-axial mass spectrometer as described in Laplante and Derome (2011). Trace metal profiles showed a polymetallic gradient in the three interconnected lakes (Supplementary Figure S1). For each lake, we measured temperature (OPA-nc: 12°C; DAS-lc: 10°C; BAR-mc: 9.9°C; LAR-hc: 11.5°C; TUR-hc: 9.5°C). Dissolved organic carbon (DOC) were determined in each sample using a total organic carbon (TOC) analyzer (Shimadzu) following the non-purgeable organic carbon (NPOC) method (Laplante and Derome, 2011).

### Water sampling

Sampling was carried out in September 2011 by collecting 6 L of water per lake at a depth of 60 cm below the surface. Water samples were sequentially filtered (3 filters per sample), first through a 47-mm poly carbonate filter with 3-micron pore size, followed by a 0.22 μm nitrocellulose membrane filter (Advantec) using peristaltic filtration (Masterflex L/S Pump System with Easy-Load II Pump Head; Cole-Parmer, Vernon Hills, IL, USA). Duplicates of the 0.22 μm filter were placed into cryotubes at −80°C.

### DNA extraction and metagenome sequencing

Filters duplicates were pooled, then genomic DNA was extracted as described by Laplante et al. (2013). Library preparation (TruSeq DNA Illumina) of paired-end reads (2 × 100 bp read length) was performed by the McGill University/Genome Quebec Innovation Center for whole metagenomic shotgun sequencing using a HiSeq™ 2000 Sequencing System. A total of 30 Gbps were obtained and the sequencing data summary is shown in Supplementary File S2. The sequence files are available from the Sequence Read Archive (http://www.ncbi.nlm.nih.gov/sra), *BioProject ID*: PRJNA449990.

### Bioinformatic and statistical analysis

#### Reads-based approach (Figure 1)

To first discard methodological biases including sequencing artifacts, we pre-processed data for quality filtering, chimeric sequences, homopolymers, and short reads (cutoff: 50 bp) using the Nesoni Clip tool (https://github.com/Victorian-Bioinformatics-Consortium) version 0.133. Overall, the quality of forward reads (R1) was better than reverse reads (R2). This difference is related to sequencing quality decrease over the length of reads, in addition to the loss of enzymatic specificity overtime in the paired-end platform technology. Base calling quality was selected at a Phred or Q score of 33 (Supplementary File S2). FLASH software v1.2.11 (Magoč and Salzberg, 2011) was used with default parameters (10–65 bp overlapping window) to merge paired-end reads.

**Figure 1:**
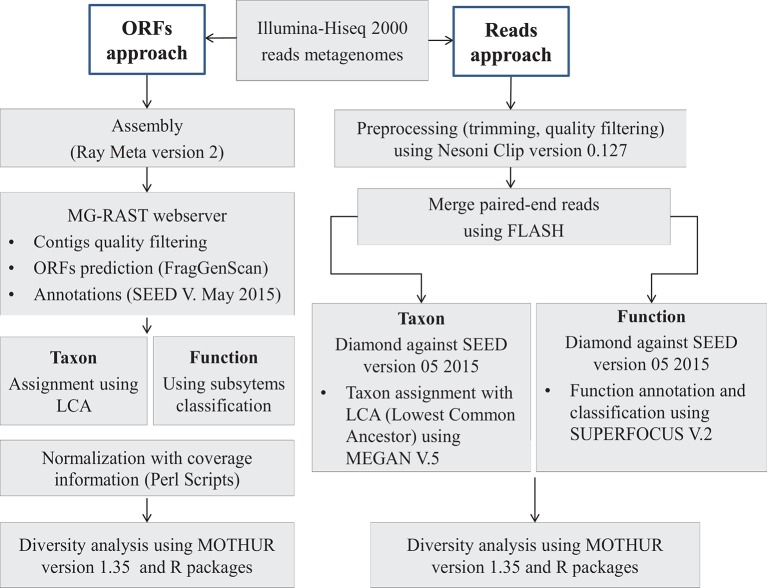
Bioinformatics analysis pipeline. Two approaches were developed for this work. With the ORF-based approach, *de novo* assembly was performed on raw reads data using *Ray Meta* software. Then, the predicted ORFs (Open Reading Frames) were annotated using Diamond similarity research tool against SEED, which is a curated database. With the reads-based approach, merged (with FLASH algorithm) and filtered reads (with Nesoni) (length ~200 pb in average) were annotated using the same tool as the ORF-based approach, Diamond algorithm and SEED database. For both approaches, we used the lowest common ancestor (LCA) algorithm in the taxonomic assignment and the subsystems hierarchy in function classification. Diversity measures were computed using mothur software.

As a second step, following the selection of good quality reads for all five metagenomic samples, a sequence similarity search was performed against the SEED database (Version: May 2015) (Overbeek et al., 2014) using Diamond v0.7.9.58 (Buchfink et al., 2015). The taxonomic content of each sample was assigned using the Lowest Common Ancestor (LCA) method (Huson et al., 2007). Functional abundance was estimated using the SUPERFOCUS software (Silva et al., 2015) with Diamond (1e^−12^ as *p*-value, 60 identity as threshold, 30 base pairs as minimum alignment length). To cope with missing biological replicates and unequal read numbers across all five lake samples (varying from 37–80 million paired-end reads before filtration), a read subsampling approach without replacement was used instead of rarefying or simulating reads from complete genomes. Accordingly, each metagenomic sample was subsampled 12 times with an equal number of reads (1 million reads). The uniformity in terms of number of subsampled reads from all samples were largely respected as in previous studies using simulated metagenomes (Mavromatis et al., 2007; Garcia-Etxebarria et al., 2014). The 60 generated metagenomic pseudo-replicates of equal size were submitted to our custom pipeline of taxonomic and functional abundance annotations.

Thirdly, to measure alpha and beta diversity based on feature abundances, we employed the OTU concept of taxonomic units (Schloss et al., 2009). Considering each feature (genus, function) as an OTU, alpha and beta diversity were computed using Mothur software (Schloss et al., 2009). UniFrac distances based on shared and unshared features were computed for each compared pair of samples. To inspect how environmental factors impact metacommunity composition, subsampled sites were first plotted based on their feature abundances with non-metric multidimensional scaling (NMDS) using Bray–Curtis distance between samples. Hence, the OPA-nc sample was used as control reference to compute the differential abundance of genera. Then, the computed distance matrix was clustered with Ward's method based on minimum variance. Clusters of genus abundance were distinguished with different colors on the NMDS plot. Next, mixed metals metadata were projected on NMDS axes by fitting a regression model. The significance of the “regression coefficient” of the model was computed using a random permutation test (1,000 iterations). Then the regression coefficient between the randomized response and the fitted values from the model was computed. The NMDS model was run using the VEGAN package (Oksanen et al., 2016) in the R statistical environment (R Foundation for Statistical Computing, 2008). To test for a correlation between taxonomical and functional composition, a Canonical Correlation Analysis using the CCA (González et al., 2008) and mixOmics (Rohart et al., 2017) packages in R were applied. With CCA, the function-taxon cross-correlation was computed by maximizing the linear combinations between the two matrix vectors. Then a regularization function of CCA from mixOmics was used to deal with the high number of features (genus, function) compared to the low number of samples (60 subsamples). Regularization parameters (λ1 and λ2) were determined through a standard cross-validation (CV) procedure on a two-dimensional surface. The optimal value for λ was obtained by searching for the largest *CV-score* on the 2D surface that requires intensive computing time to converge for the optimal cross-validation value. Choice of canonical dimensions and graphical representation of features and samples were performed with mixOmics package.

#### ORFs-based approach (Figure 1)

To improve annotation accuracy in terms of length and coverage, an Open Reading Frame (ORF) prediction approach was used after *de novo* assembly. Collinear metagenomic reads belonging to the same genetic unit were merged into contiguous sequences (contigs). Firstly, *de novo* assemblies of raw reads were performed using the *RAY Meta* (Boisvert et al., 2012) assembler. Secondly, to explore contig features and gene contents, contigs were submitted to the MG-RAST webserver (Glass et al., 2010) and ORF prediction was conducted using the FragGeneScan tool (Rho et al., 2010). Afterwards, contigs were annotated with the BLAT tool implemented in MG-RAST against the SEED database using stringent filtering parameters (1e^−12^ as *p*-value, 85% identity as threshold, 50 base pairs as minimum alignment length). Statistical summaries of annotated contigs are available in Supplementary File S2. Customized microbial annotations from the MG-RAST webserver were improved using the RESTful API tool (Wilke et al., 2015). An additional similarity research step based on BLASTx (parameters; identity threshold of 85%, *e*-value of 10^−12^ and minimum alignment length of 50 base pairs) (Camacho et al., 2009) was performed on contigs against the BacMet database (Pal et al., 2014) for annotating all polymetallic resistance genes (hereafter termed PMRGs). After annotations, contig coverage information determined by the Ray Meta assembler was added to normalize abundance information. Then, both abundance matrices of taxon and function coverage (both normalized and non-normalized) were analyzed with the STAMP software using a differential proportion comparisons test (Parks and Beiko, 2010). In a second additional workflow analysis, the ORFs were locally annotated with Diamond as described above in the “Reads-based approach” section. BLAT and Diamond provided similar annotation results. To measure *alpha* and *beta* diversity within and between communities, abundance matrices were adapted for the Mothur software. At the third step, metabolic abundance was analyzed using MG-RAST metabolite annotations. The metabolic differential abundance was surveyed using iPATH (Yamada et al., 2011); this tool offers the visualization of shared and specific pathways between pairs of samples.

## Results

### Decoupling taxon-function

To investigate the impact of the polymetallic selection gradient on lake metacommunity composition, we measured the pattern of decoupling between taxon and function along the contamination gradient of the five lakes. We hypothesized that taxon-function decoupling pattern is an adaptive response of lake metacommunities. To detect this pattern, we performed two independent analyses: (i) taxonomic structure vs. functional diversity and (ii) canonical correlation of taxon and function.

### Detangled taxonomic structure and function diversity

**Figure 2:**
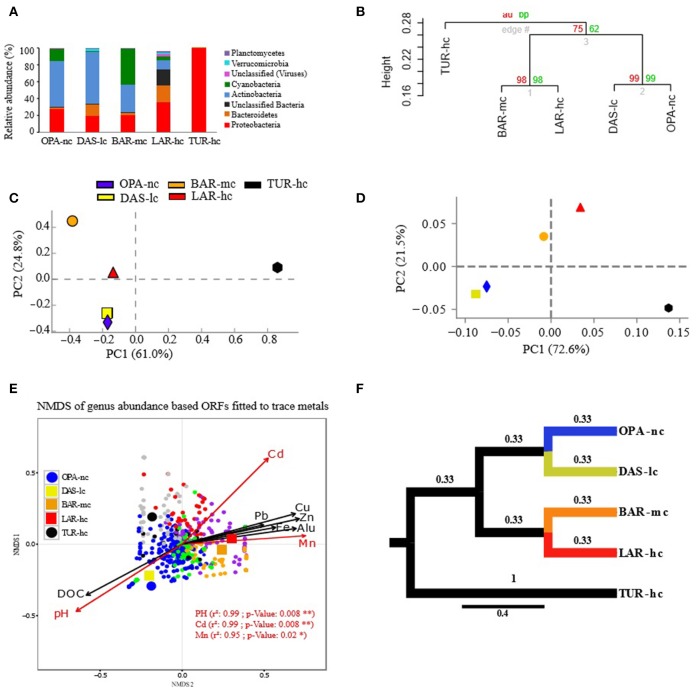
Composition of metacommunities based on the ORF approach. **(A)** Metacommunity composition (y-axis) is shown in stacked bars for each lake metagenome (x-axis). Only phyla with relative abundance (RA) greater than 1% are shown. **(B)** Hierarchical clustering of samples based on genus RA using Ward's method and Bray–Curtis dissimilarity distance, bootstrap AU (Approximately Unbiased) *p*-value and BP (Bootstrap Probability) values are shown on the nodes. **(C,D)** Principal Component Analysis (PCA) of samples based on genus RA with different annotation parameters of alignment length cutoffs (50 pb in c and 30 bp in d) and identity threshold (85% in **C** and 60% in **D**). **(E)** NMDS of genera abundance fitted to trace metals was performed with Bray–Curtis distance, three dimensions were *a priori* defined for distance rank ordination and stress value was below 0.05. Cadmium (Cd), Manganese (Mn), and pH significantly fitted with NMDS axes are highlighted in red. NMDS loadings (NMDS1, NMDS2), and *P*-value of correlation *r*^2^ of trace metals were reported in Supplementary File S6. Each small dot represents the ordinated genus, while each large point represents the lake communities' samples using a circle for OPA-nc in blue and the control TUR-hc in black, and the connected lakes are illustrated with squares (LAR-hc in red, BAR-mc in orange and DAS-lc in yellow). Genus plot coordinates, clusters and dot labels are shown in Supplementary File S4. **(F)** Tree based Unifrac distance computed with mothur is indicated by branch lengths. All these results were obtained using the ORF based approach with 85% identity threshold, *e*-value of 10^−12^, minimum alignment length of 50 base pairs, and the lowest common ancestor (LCA) algorithm for taxonomic assignment. OPA-nc (Opasatica Lake) is the negative control; DAS-lc (Dasserat Lake) is low polluted; BAR-mc (Arnoux Bay) is medium polluted; LAR-hc (Arnoux Lake) is highly polluted, and TUR-hc (Turcotte Lake) is the positive control of contamination.

**Figure 3:**
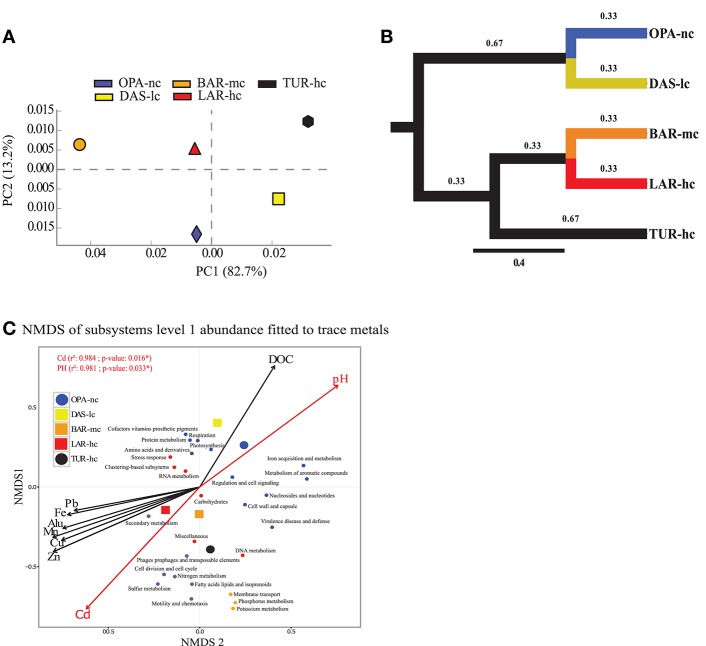
Function abundance classification based on ORF approach. Composition analysis of metacommunity functions based on relative abundance (RA) of subsystems using principal component analysis (PCA) **(A)**, tree based Unifrac distance **(B)** and NMDS **(C)** identified the same pattern. **(A)** PCA figure was obtained from STAMP software (Parks et al., 2014). **(B)** NMDS (three *a priori* predefined dimensions projected into two dimensions, stress value < 0.05, Bray–Curtis distance) axes of all annotated subsystems level 1 fit significantly with Cadmium (Cd), and pH using the ORF approach. In d, each small dot represents a subsystem, while the large dot does represent the lake metagenome indicated with a circle for the negative control lake (OPA-nc) in blue and the positive control lake (TUR-hc) in black. The connected lakes are illustrated with squares (LAR-hc in red, BAR-mc in orange and DAS-lc in yellow). NMDS loadings (NMDS1, NMDS2), and *P*-value of correlation *r*^2^ of trace metals were reported in Supplementary File S6. Subsystems plot coordinates, clusters, and dot labels are resumed in Supplementary File S4. **(C)** Tree based Unifrac distance computed with mothur is indicated by branch lengths In the ORF based approach the following parameters were strictly respected; 85% identity threshold, *e*-value of 10^−12^, minimum alignment length of 50 base pairs, and the lowest common ancestor (LCA) algorithm for taxonomic assignment. OPA-nc (Opasatica Lake) is the negative control; DAS-lc (Dasserat Lake) is low polluted; BAR-mc (Arnoux Bay) is medium polluted; LAR-hc (Arnoux Lake) is highly polluted, and TUR-hc (Turcotte Lake) is the positive control of contamination.

According to *alpha-diversity* analysis, the highest value of community *richness (chao index)* at the genus level was recorded in BAR-mc (OPA-nc: 126.8, DAS-lc: 115.07, BAR-mc: 212.6, LAR-hc: 121.66, TUR-hc: 68). In contrast to richness, community *evenness (Shannon index)* was lowest in TUR-hc (0.116), intermediate in OPA-nc (2.24), gradually decreasing along the metallic gradient from DAS-lc (2.85) < BAR-mc (2.58) < LAR-hc (2.47). However, community *evenness* of functions (OPA-nc: 2.36, DAS-lc: 2.32, BAR-mc: 2.92, LAR-hc: 2.62, TUR-hc: 2.87) was higher in BAR-mc, LAR-hc and TUR-hc then OPA-nc and DAS-lc. Then, *beta-diversity* analysis at the genus level (Figure 2F) revealed two patterns of structural convergence: (i) between the two independent lake communities, namely the unpolluted control (OPA-nc) and the low polluted lake (DAS-lc); (ii) between the interconnected BAR-mc-LAR-hc and the polluted control TUR-hc communities. Concerning functional diversity distribution, *beta-diversity* of all subsystems (Figure 3B) revealed two convergent patterns: (i) between the polluted control (TUR-hc), the highly-polluted gradient lake (LAR-hc), and the medium-polluted lake (BAR-mc); (ii) between the independent lake communities, namely the low-polluted DAS-lc and the negative control (OPA-nc) communities.

**Figure 4:**
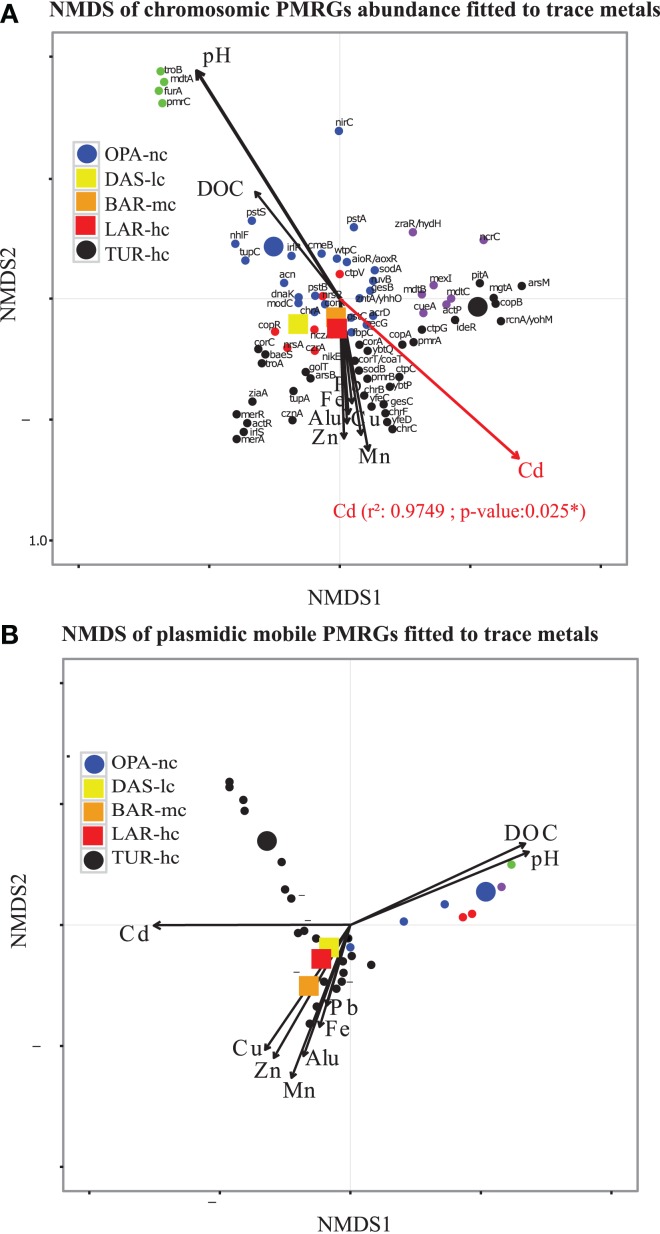
Polymetallic resistance genes (PMRG) abundance correlation with trace metals. **(A)** For PMRG on chromosomes (72 genes), Cadmium (Cd) was significantly correlated with NMDS axes and it was the main explanatory factor of abundance variation of these genes between metacommunities. **(B)** NMDS axes based on relative abundance of PMRG located on plasmids (27 genes) do not significantly fit with any trace metal arrows. This NMDS analysis was performed with Bray–Curtis distance, three dimensions were *a priori* defined for distance rank ordination and stress value was below 0.05. NMDS loadings (NMDS1, NMDS2), and *P*-value of correlated trace metals are reported in Supplementary File S6. Each small dot represented an individual PMRG, while each large point represents the lake communities' samples using circles for OPA-nc in blue and the control TUR-hc in black, and the connected lakes were illustrated with squares, LAR-hc in red, BAR-mc in orange and DAS-lc in yellow. PMRG plot coordinates, clusters and dot labels are shown in Supplementary File S4. Thresholds of 75% of identity, minimum alignment length of 50 base pairs and *e*-value of 10^−12^ parameters were strictly respected. PMRG were annotated by performing Blastn of ORFs against BacMet database using Diamond software.

### Canonical correlations of taxon and function

**Figure 5:**
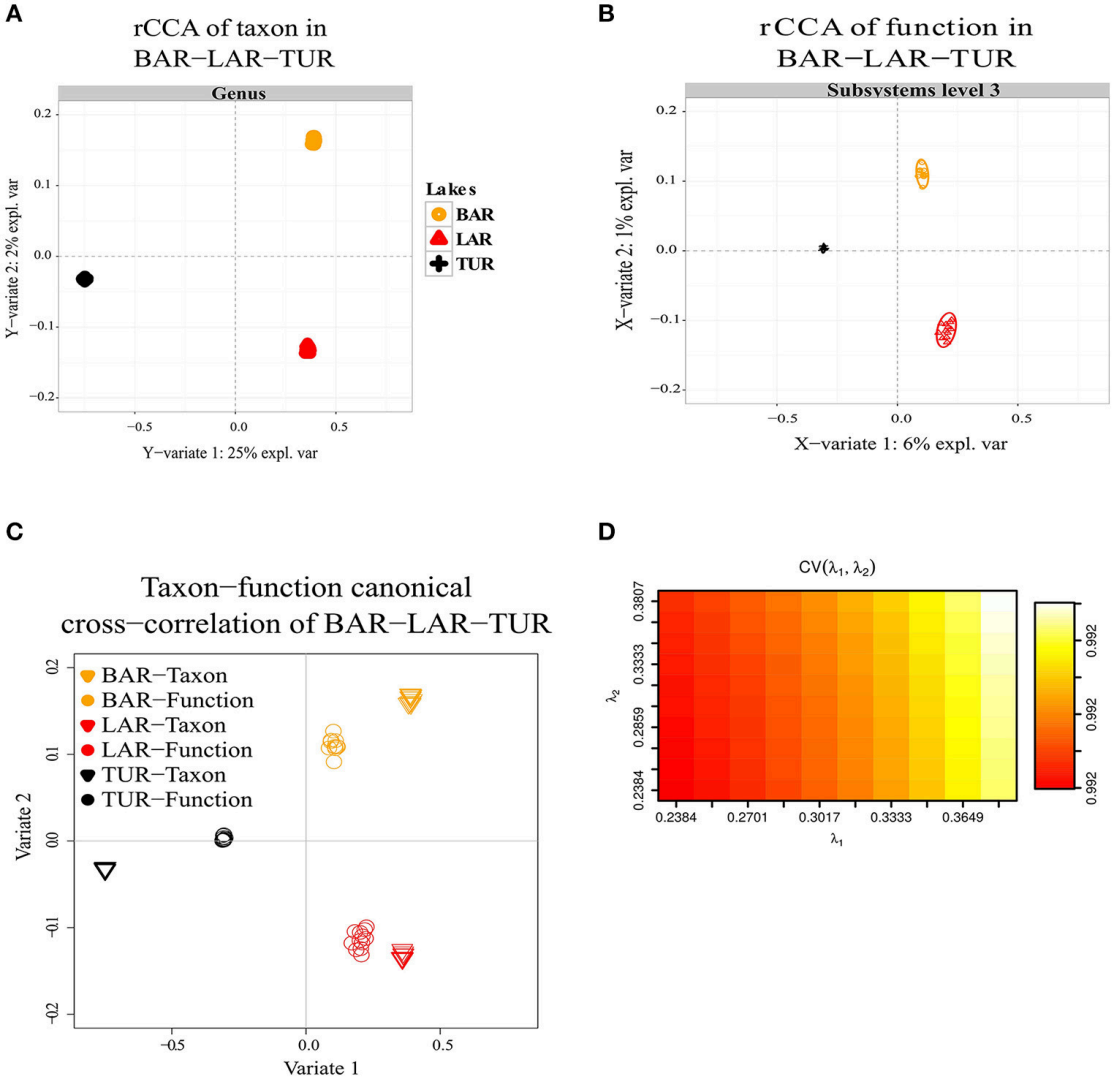
Decoupling of taxon and function between metacommunities based on the subsampled reads approach. **(A–D)** Regularized canonical correlation analysis (rCCA) performed on BAR-mc, LAR-hc, and TUR-hc. **(A)** rCCA of a taxon (relative genus abundance) showed 25% of the explained variance on the first canonical component. **(B)** rCCA of function (subsystems level3 relative abundance) showed only 6% of the explained variance on the first canonical component. **(C)** The canonical cross-correlation of taxon-function identified a decoupling pattern. **(D)** Cross-validation score converged to a maximum value of 0.99 when regularization parameters λ1 and λ2 were both fixed at 0.375.

**Figure 6:**
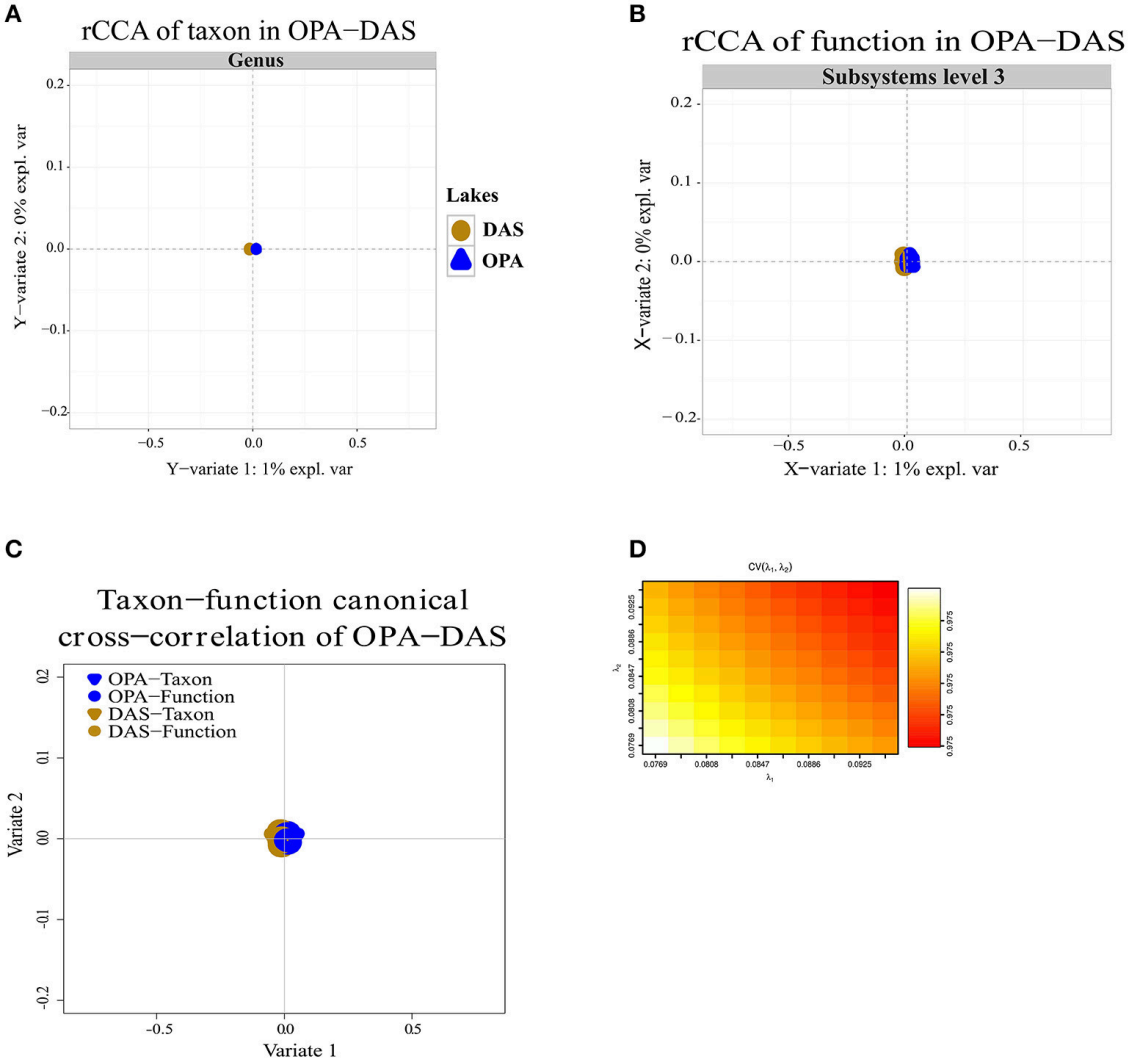
Coupling of taxon and function between metacommunities based on the subsampled reads approach. **(A–D)** Regularized canonical correlation analysis (rCCA) performed on OPA-nc, and DAS-lc. **(A)** rCCA of the taxon (relative genus abundance) showed 1% of the explained variance on the first canonical component. **(B)** rCCA of function (subsystems level3 relative abundance) also showed 1% of the explained variance on the first canonical component. **(C)** The canonical cross-correlation of taxon-function identified a coupling pattern between taxon and function. **(D)** Cross-validation score converged to a maximum value of 0.975 when regularization parameters λ1 and λ2 were both fixed at 0.0925. The rCCA method was applied using mixOmics and CCA package in R.

Regularized canonical correlation analysis (rCCA) of function (subsystems level 1, 2, 3) with taxon was assessed using a maximal cross-validation criterion (see Materials and Methods). To detect linear combinations between function and taxon we separately performed the same rCCA analysis for OPA-nc/DAS-lc, and then for BAR-mc/LAR-hc/TUR-hc. For OPA-nc/DAS-lc (**Figures 6A**–**D**), we found a maximum variance of only 1% explained by the first axis computed from the taxon covariance matrix, and 1% explained by the first canonical correlation principal component computed from the function subsystems level 1 (results not shown) covariance matrix, even when we tested the canonical model at the most accurate hierarchical functional resolution (subsystems level 3). Supported by a high cross-validation score (0.975), this result suggested a strong coupling between taxon and function. Conversely, in BAR-mc/LAR-hc/TUR-hc (Figures 5A–D) we found the first axis explained 25% of the variance computed from the taxon covariance matrix, and 3% (Subsystem level 1) to 6% (subsystems level 3) explained by the first canonical axis computed from the functional covariance matrix. This result, supported with a high cross-validation score (0.99), revealed a weak correlation between taxon and function, thus suggesting a strong taxon-function decoupling. Using the first two canonical axes, in BAR-mc, LAR-hc, and TUR-hc, a clear separation was observed between taxon and function (Figure 5C), while the axes are superimposed in OPA-nc and DAS-lc (Figure 6C).

### Taxonomic variation signatures

The metacommunity composition analysis emphasized three major patterns marked by abundance shifts within and between *Proteobacteria, Cyanobacteria* and *Actinobacteria* phylum (Figure 2A). In the first pattern, ***Proteobacteria*** mostly dominated by *Betaproteobacteria* (Supplementary File S3) reached a higher relative abundance in the highly-polluted (hc) lakes TUR-hc (99%) and LAR-hc (35%) compared to the moderately-polluted (mc) lake BAR-mc (20%), the least-polluted (lc) lake DAS-lc (19%), and the unpolluted (nc) lake OPA-nc (27%). At the genus level, *Polynucleobacter, unclassified Burkholderia*, and *Burkholderia* were the most dominant within polluted lakes TUR-hc, LAR-hc, and BAR-mc, respectively, while *Polaromonas* was the most dominant in DAS-lc and OPA-nc (Supplementary File S3). In the second pattern, ***Actinobacteria*** were the most dominant phylum (Supplementary File S3) in less polluted lakes [OPA-nc (53%) and DAS-lc (62%)] and their relative abundance gradually decreased in more polluted lakes [BAR-mc (33%), LAR-hc (10%) and completely disappeared in TUR-hc], mainly for the five most abundant genera: *Streptomyces, Frankia, Mycobacterium, Kribbella*, and *Nocardioides* (Supplementary File S3). In the third pattern, *Cyanobacteria* (Supplementary File S3) were abundant in OPA-nc (15.4%) and BAR-mc (42%), and much less frequent in LAR-hc (4.4%), DAS-lc (0.2%), and TUR-hc (< 0.01%). At genus level, *Synechococcus* was most dominant, accounting for 98% and 92% of *Cyanobacteria* genera in OPA-nc and DAS-lc, respectively. In contrast, distinct *Cyanobacteria* genera were dominant in polluted lakes: the filamentous *Anabaena* in BAR-mc, unclassified *Cyanobacteria* in LAR-hc, and both the diazotrophic *Cyanothece*, and the filamentous *Anabaena* in TUR-hc.

Lake metacommunity abundance shifts were further documented using bootstrapped hierarchical classification and PCA. At the genus level, both methods showed similar pattern of clustering with high statistical support (bootstrap values above 75%; more than 95% of explained variation by the first two PCA components), with BAR-mc, LAR-hc, and TUR-hc grouped separately from OPA-nc, DAS-lc (Figures 2B–D).

### Role of trace metals in taxonomic variation signatures

NMDS analysis based on ORFs (Figure 2E) revealed interesting relationships (significant R-squared indicating regression model's goodness of fit) between taxonomic abundance and different factors such as pH, DOC and trace metals (mainly Cadmium). OPA-nc and DAS-lc were significantly correlated with DOC and pH axes, while all other sites exposed to polymetallic gradient (BAR-mc, LAR-hc, and TUR-hc) were significantly correlated with trace metals axes (Figure 2E). To further analyze the link between abundance shifts at different taxonomic ranks and the trace metal gradient, the same NMDS analysis was performed using the ORFs approach. The abundance of *Proteobacteria* (Supplementary Figure S3a), *Actinobacteria* (Supplementary Figure S3b), and *Cyanobacteria* (Supplementary Figure S3c) were studied separately. NMDS analyses of abundance shifts at the genus level revealed significant correlations with different metal axes, pH and DOC. The shifts in composition within lake metacommunities were not explained by the same factors. For example, variation in the abundance of *Proteobacteria* among lakes was mainly explained by Cd, pH, Mn, Alu, while Cd and Fe explained variation in the abundance of *Actinobacteria*, and Alu and Mn were the main factors explaining variation in the abundance of *Cyanobacteria* among lake metacommunities.

### Function variation signatures

Our results showed 6,801 annotated functions from all communities distributed into 988 subsystems in level 3, 192 subsystems in level 2, and 28 subsystems in level 1 (see sheet 2 in Supplementary Figure S5). At the first level (see Supplementary Figure S5 and Supplementary File S5), our results of cross-metagenomes comparison suggested that the relative abundance of “Photosynthesis,” “Cofactors, Vitamins, Prosthetic Groups, Pigments,” and “Respiration” subsystems was significantly highest in OPA-nc while the “Stress response” was the lowest in this lake. However, Subsystems of “RNA metabolism,” and mobile elements (Phages, prophages, plasmids, and transposable elements) showed the highest abundance in BAR-mc, followed by LAR-hc and TUR-hc, and low abundance in OPA-nc and DAS-lc. Furthermore, the relative abundance of the “carbohydrates” subsystem decreased gradually in all lakes except from DAS-lc to OPA-nc (Supplementary File S5). Interestingly, among the 28 subsystems (Level 1), four subsystems “Nitrogen metabolism,” “Cell cycle and division,” “Sulfur metabolism,” “and “Motility and Chemotaxis” decreased gradually along the contamination gradient. In addition, three subsystems (Phosphorus and Potassium metabolism, Membrane transport) were absent in LAR-hc and showed specific profiles of low abundance (Supplementary File S5) varying between 0.2 and 3.8% in BAR-mc and TUR-hc. For multiple subsystems in Level 1 (*n* = 12), no gradual abundance variation was observed. However, at a deeper resolution, many important functions related to metals transport and resistance from the “Virulence defense and disease,” “Membrane transport,” and “Iron acquisition and metabolism” subsystems showed few gradual (i.e., Cobalt-Zinc-Cadmium resistance) abundance profiles and high specific abundance per lake (Supplementary Figure S7). At the functional level, variation abundance was detectable within all subsystems where three profiles of abundance variation were observed from OPA-nc to TUR-hc: (i) profile 1 (FP1) represents gradual function abundance decrease (106 functions) along the contamination gradient (Supplementary File S5 and Supplementary Figure S10), (ii) profile 2 (FP2) represents gradual function abundance increase (123 functions) along the contamination gradient Supplementary File S5 and Supplementary Figure S10, and (iii) profile 3 (FP3) represents specific functional abundance (Supplementary File S5 and Supplementary Figure S11) in control negative OPA-lc (167 functions), or in polluted lakes (225 functions). These functional profiles were not necessarily observed in one subsystem, but rather multiple profiles were detectable within one subsystem (Supplementary File S5). For example, under the “Virulence, Disease and Defense” subsystem, we observed all these profiles with functions related to metal resistance FP2 (i.e., Cobalt-zinc-cadmium CzcA protein, Cation efflux system protein CusA), and FP1 (i.e., Magnesium and cobalt efflux protein CorC), and FP3 (i.e., Copper homeostasis) OPA-nc (see Virulence subsystem in Supplementary File S5). However, functions related to mobile genes and HGT agents (Supplementary Figure S8) were significantly more abundant in polluted lakes (e.g., Gene transfer agent proteins, conjugative transfer proteins, DNA repair, CRISPR associated proteins, integrons). Classification of functional abundance (subsystem levels 1, 2, 3) identified two independent clusters. The first cluster grouped BAR-mc, LAR-hc and TUR-hc, and the second grouped DAS-lc and OPA-nc (Supplementary Figures S5, S6). Similar topologies were obtained using both approaches: ORF (Supplementary Figure S4b) and reads subsampling (Supplementary Figures S4c,d). PCA analysis based on the ORF approach produced the same results, where at least 71% of variance was explained on the first PC for all subsystem function levels. We only presented a PCA plot for subsystems abundance in level 1, where more than 82% of variation in functional abundance was explained by the first component (Figure 3A). At the metabolic level, analysis of enzymes abundance profiles cross-metagenomes showed different topology which was a dichotomy between OPA-nc and all others pollution gradient lakes (See Supplementary File S7 and Supplementary Figure S9).

### Role of trace metals in function variation signatures

NMDS analysis of functional abundance highlighted two main patterns of correlation (significant R-squared indicating regression model's goodness of fit) with metadata (Figure 3C). First, BAR-mc, LAR-hc, and TUR-hc were correlated with Cadmium axis (*p* ≤ 0.05). Second, OPA-nc and DAS-lc were correlated with pH axis (*p* ≤ 0.05). The same analysis performed on the subsystems in level 2 (192 functional modules) suggested a significant contribution of all studied factors (results not shown). At the finest functional level, lakes ordination based on the NMDS of polymetallic resistance genes (PMRG)s abundance showed a fit with the cadmium concentration gradient (Figure 4), where DAS-lc was ordinated near BAR-mc and LAR-hc. In NMDS analysis of PMRGs located on chromosomes (Figure 4A), only Cadmium played a significant role in explaining abundance variation. Similarly, the NMDS analysis of PMRGs located on plasmids provided the same classification profile even though they do not fit significantly with any metal traces (Figure 4B).

## Discussion

### Decoupling taxon-function as a signature of adaptive strategies

Comparing the compositional signatures of taxon and function, we observed that relative shifts in taxon abundance could only partially predict the impact of metallic toxicity on metacommunity structure (see section Role of Trace Metals in Taxonomic Variation Signatures). By considering the signatures of functional abundance of the subsystems explained by pH and Cadmium in polluted lakes, we could more accurately predict the impact of metallic contaminants on ecosystem services of lake metacommunities. In this respect, the contamination gradient explained much variation in community function structure and provided a powerful way to further assess the relationship between the distribution of functional abundance and selective pressure, which may increase gradually with the expelled AMD flow over time. The impact of the selection gradient on lake metacommunity composition was tested through two independent analyses, first using diversity measures, and second by detecting taxon-function decoupling patterns. Alpha taxonomic diversity suggest a switch in BAR-mc, while the gradual decrease in evenness based both taxon and function in OPA-nc: (2.2^t^; 2.3^f^), DAS-lc (2.8^t^; 2.3 ^f^), BAR-mc (2.5^t^; 2.9^f^), LAR-hc (2.4^t^; 2.6^f^), TUR-hc (0.1^t^; 2.8^f^) could be a potential consequence of composition homogeneity in community type (e.g., *Proteobacteria* in TUR-hc). Indeed, this observation may be related to the low complexity in AMD communities previously documented for the same lake system (Laplante and Derome, 2011; Laplante et al., 2013), and for other AMD metacommunities (Allen and Banfield, 2005; Huang et al., 2016).

The rCCA analysis allowed for the detection of significant spatial correlation between taxon and function in OPA-nc/DAS-lc, reflecting a coupling between taxon and function. In these unpolluted lakes, as mentioned above, NMDS analysis showed that environmental factors (Cadmium, pH, and DOC) explained variation in the overall taxonomic and functional composition. At high resolution (subsystems level 2, 3) NMDS showed a slight difference between OPA-nc and DAS-lc, but we cannot unequivocally associate these variations to trace metal ratios. We may have missed other explanatory environmental and chemical variables (i.e., NFigure_2_, NO_3_, SO_4_, PO_4_), or the potential variation resulting from neutral ecological process, drift or random reproduction as observed in wastewater habitats (Ofiteru et al., 2010). Such coupling is not necessarily absolute but partial, owing to the presence of some differentiated sub-communities performing the same ecosystem services. In pristine natural conditions (without stressful anthropogenic inputs), coupling between taxon and function was observed in freshwater lakes (Langenheder et al., 2005; Debroas et al., 2009), and decoupling was observed in oceanic bacterial communities from contrasted environments (Louca et al., 2016b).

Overall, in the present study, we found that functional variation between polluted and unpolluted lakes was better explained by environmental factors than taxonomic variation between and within functional groups. Concerning the three lake communities facing exposure to a polymetallic gradient (BAR-mc/LAR-hc/TUR-hc), the explained variance between taxon (25%) and function (6%) strongly suggests a decoupling between taxa and functions. The shared functions in these three polluted lakes reflect a convergent pattern, which in turn could be interpreted as a predictive signature of the ecosystem service's impairment associated with acid mine lake water. This conclusion is further supported by the NMDS results, where the distribution of polluted lakes fitted closely to Cadmium. In addition to rCCA, when comparing tree topologies of structure and function (Figures 2F, 3C), we detected additional patterns of taxon-function decoupling, like the PCG. Such an approach offers interesting insights into the adaptive strategies used by metacommunities facing long-term exposure to polymetallic pollution. Often interpreted as an indicator of HGT in natural communities (Ram et al., 2005; Green et al., 2008; Burke et al., 2011; Louca et al., 2016a,b) and AMD communities (Navarro et al., 2013; Devarajan et al., 2015; Chen et al., 2016; Hemme et al., 2016), taxon-function decoupling may provide evidence for selective pressure on microbial communities (e.g., exerted by metallic exposure). Indeed, as mentioned above, multiple proteins playing a role in HGT, such as cassettes of integrons and transposable elements, were present in polluted lakes, and absent in an unpolluted lake (OPA-nc). We observed more than 14 mobile PMRGs located on plasmids, and only two PMRGs on both plasmids and chromosomes. The plasmid location of these PMRGs indicates that bacterial conjugation may be a vector for HGT. Interestingly, a heatmap of abundance clustering from chromosomal and plasmid PMRGs (figure not shown) produced a similar topology of functional profiles (i.e., OPA-nc; DAS-lc-BAR-mc; LAR-hc-TUR-hc).

Evolutionarily speaking, such taxon-function decoupling patterns are expected to be signature of adaptation within communities between closely, but also distantly, related bacterial strains. Consequently, community composition in BAR-mc, LAR-hc or TUR-hc may have independently evolved via HGT events of resistance and regulatory genes. According to functional abundance results, the potential occurrence of HGT is higher in LAR-hc and TUR-hc compared to BAR-mc, which is closer to DAS-lc and OPA-nc in terms of functional distribution. A subset of adaptive beneficial transferred genes is expected to reach fixation (Lind et al., 2010), but the long term metallic contamination may have funneled the “metal resistance gene pool” into different evolutionary trajectories due to the mounting selective pressure.

### Taxonomic adaptive signatures

In this study, the overall taxonomic variation suggests three salient patterns of abundance distribution. First, a “composition gradient” pattern constituted three shifts in taxonomic structure: (i) high abundance of *Proteobacteria* in polluted sites (TUR-hc, LAR-hc; BAR-mc), (ii) high abundance of *Actinobacteria* in unpolluted sites (OPA-nc, DAS-lc), (iii) intermediate levels of *Cyanobacteria* in all sites, with *Nostocales* being abundant in polluted lakes and *Chroococcales* abundant in unpolluted lakes (Supplementary File S3). Second, a “community type” pattern suggests that the overall metacommunity exhibited compositional shifts along the five lakes from wide (phylum) to narrow (genus) taxonomic levels. Third, a “taxonomic convergence” pattern highlights parallel changes of community taxonomic structure, thus confirming previous results based on semi-quantitative and quantitative studies (Laplante and Derome, 2011; Laplante et al., 2013).

To further reinforce the taxonomic composition analysis, we examined genera abundance and ORF distributions. Similar ratios of ORFs/Genus were observed in the five studied metagenomes (Supplementary Figure S2). The number of annotated ORFs in all metagenomes was comparable. Furthermore, random subsampling analysis without replacement produced similar results (slightly different in topology) compared to the ORFs approach, with remarkable clustering fidelity of subsampled replicates from each metagenome (Supplementary Figure S4a). Here, the subsampling approach revealed consistency in the molecular signal of each lake. We acknowledge that the subsampling approach used in our analysis cannot replace real biological replications, but it is rather an indicator of the metagenomic data robustness to the metacommunity structure.

To understand the sources of variation in contributing to the three major shifts of relative abundance in community type, combined NMDS and correlational analyses were performed for each pattern of taxonomic variation. First, the *Proteobacteria* genus distribution of eight predefined clusters (Supplementary File S4) showed that abundance variation between communities was mainly explained by synergistic interactions of Cd, pH, Mn, and Alu (Supplementary Figure S3a). According to previous studies, *Proteobacteria* were among the most abundant phyla in acid mine water (Laplante et al., 2013; Streten-Joyce et al., 2013) and in freshwater lake sediments polluted by “heavy metals” (Ni et al., 2016). Second, in contrast to *Proteobacteria*, our results divided *Actinobacteria* into four genus abundance clusters (Supplementary File S4) constrained by two main and opposite explanatory factors, Cd and Fe (Supplementary Figure S3b). In fact, the most abundant *Actinobacteria* genera (*Streptomyces, Frankia, Mycobacterium*), which varied between polluted and unpolluted lakes, fall in the same abundance cluster (see *Actinobacteria* in Supplementary File S4). Indeed, some *Actinobacteria* (e.g., *Streptomyces*) strains are known to have different metal-resistance profiles (Álvarez et al., 2013). Interestingly, strains like *Mycobacterium* were able to transport and uptake Cd (Dimkpa et al., 2009). On the other hand, *Cyanobacteria* abundance showed different patterns of abundance in polluted and unpolluted lakes (Supplementary File S4 and Supplementary Figure S3c) suggesting that *Chroococcales* (*Cyanothece, Microcystis, Synechocystis, Thermosynechococcus*) and *Synechococcales* (*Synechococcus, Prochlorococcus*) are much more affected by trace metals compared to the *Nostocales (Anabaena, Aphanizomenon, Cylindrospermopsis, Dolichospermum, Nodularia, Nostoc, Raphidiopsis)*. Although Cd was not identified here as a significant explanatory factor, diverse strains of *Nostocales* were documented to have the capacity to adsorb Cadmium (Pokrovsky et al., 2008) and trace metals (Mota et al., 2015). Interestingly, the sudden break of *Nostocales* lineages (Supplementary File S3) between the connected lakes DAS-lc, BAR-mc and LAR-hc is potentially related to resistance thresholds to trace metals, as higher levels become toxic to *Synechococcus* (Ludwig et al., 2015). Furthermore, the high relative abundance of *Chroococcales* and *Cyanobacteria* in OPA-nc and DAS-lc is potentially related to their role in photosynthesis and DOC mineralization (Bittar et al., 2015). Overall, our results show that metallic toxicity impacts metacommunity structure and provides a partial explanation for the relative shifts in abundance found in the lakes we studied. The dominance of *Proteobacteria* in over polluted communities confirms the result previously observed in the same lake system (Laplante et al., 2013), and from various acid mine waters in the world (Almeida et al., 2008; Hemme et al., 2010; Kuang et al., 2013; Stankovic et al., 2014; Wang et al., 2015).

### Functional adaptive signatures

At the general level (subsystems level 1), only four subsystems showed gradual variation. At the function level, our results suggest deterioration in ecosystem services along the contamination gradient, as relative abundance of functional modules in 18 subsystems such as “*Carbohydrates,” “Photosynthesis*,” “*Cell division and cycle*,” “*DNA metabolism*,” and “*Respiration*” decrease gradually. However, under “*Virulence defense and disease*” and “*Membrane transport*” subsystems (level 1), many important metals transport and resistance functions (i.e., Cobalt-Zinc-Cadmium resistance) increased between OPA-nc and other lakes (Supplementary Figure S7). These profiles of gradual changes were less observable at the general level (subsystems level 1), and more detectable at the functional level resolution of many subsystems. The gradual decrease and increase in relative abundance proportions was clearly observed at le the lowest molecular function (i.e., *Photosynthesis* functions) along the polymetallic gradient. Overall, variation in the functional composition of metacommunities suggests convergence between BAR-mc/LAR-hc and TUR-hc, two geographically distant and independent lakes affected by independent AMD sources.

In contrast to the community classification based on taxonomic composition, BAR-mc is functionally closer to LAR-hc-TUR-hc than OPA-DAS. NMDS of functional composition, community hierarchical clustering, and PCA analysis all find the same classification results. Cadmium and pH were the main factors explaining functional composition variability among lakes. However, independent analysis performed on both PRMGs and enzymatic functions abundance showed that DAS-lc fitted within the polluted lakes (BAR-mc-LAR-hc-TUR-hc) instead of OPA-nc. PMRGs located on plasmids (Figure 4B) were differentiated from those located on chromosomes (Figure 4A) since plasmid genes are known to house more adaptive genes acquired via bacterial conjugation (Li et al., 2015). Only two experimentally confirmed genes (*copA* and *actP)* were found in both plasmids and chromosomes. *CopA* is involved in silver/copper export and homeostasis (Cha and Cooksey, 1991; Outten et al., 2001; Banci et al., 2003; Behlau et al., 2011). Acetate Permease (ActP) controls copper homeostasis in rhizobium preventing low pH-induced copper toxicity (Reeve et al., 2002). NMDS analysis based on Chromosomal PMRG abundance revealed that Cadmium plays a significant role (*p* ≤ 0.05) in shaping the differential abundance of these genes. Alternatively, analysis of plasmid PMRGs did not highlight any significant fit with metal axes (Figure 4B), owing to the low number of annotated PMRGs on plasmids. Using OPA-nc as an unpolluted reference in our comparative framework, differential metabolic abundance variation revealed an erosion of biosynthesis pathways along the contamination gradient (results of compared pathways not shown). Eroded metabolic functions were associated to degradation of aromatic compounds, amino acid biosynthesis, and carbohydrates, thus leading to the loss of major bacterial mediated ecosystem services. As bacterial communities experienced a consistent metallic stress over 60 years of mining activities, many functions associated with ecosystem services likely became energetically too expensive to be maintained. Such a selective environment may have led to community specialization. Community specialization has recently been demonstrated in soil AMD communities (Volant et al., 2014) and natural freshwater communities (Pernthaler, 2013; Salcher, 2013; Pérez et al., 2015). In summary, the two main elements (or factors) that explained the majority of the functional variation between polluted vs. unpolluted communities were pH and Cadmium concentration. Nonetheless, other metal trace gradients offered partial explanations for functional variation.

## Conclusions

In this study, we examined adaptive signatures within natural lacustrine microbial communities living under a gradient of selective pressure induced by trace metal contamination from over 60 years of mining. Using a metagenomic approach based on whole genome shotgun sequencing, we identified a convergence in both taxonomic and function responses, thus providing evidence for genotypic signatures of adaptive evolution. Strong selective pressure may drive overall taxon-function decoupling, which may reflect the occurrence of gene loss and HGT induced by AMD gradient, or the result of strong selection exerted on existing strains possessing the necessary resistance genetic background. This study remains a preliminary assessment of decoupling phenomenon and further studies are eventually needed to understand in a deeper manner the nature of convergence between unpolluted environments vs. polluted environments in a context of stress gradient. At the taxonomic scale, metacommunity composition showed marked relative abundance shifts of major phyla, but was much more marked at the genus level, suggesting a “community type” adaptation to the metallic gradient within each ecological niche. At the function scale, we observed the erosion of metabolic pathways along the metallic gradient despite the higher abundance of functional categories like stress response, regulation, protein metabolism, and metallic resistance in polluted lakes compared to unpolluted lakes. Investigating the relationship of both taxonomic and functional signatures, we detected a decoupling pattern between taxon and function in polluted lakes as an indicator of adaptation potentially via HGT. These results suggest, for the first time, a decoupling pattern of taxon-function within natural communities adapted to a gradient of polymetallic contamination. This decoupling pattern highlights the gap between microbial biodiversity and ecosystem services in polluted environments.

## Author contributions

ND conceived the experiment. P-LM conducted the experiment. ML performed the data assembly. BC produced and analyzed the results. BC and ND wrote the manuscript. This project was under supervision of ND. All authors reviewed the manuscript.

### Conflict of interest statement

The authors declare that the research was conducted in the absence of any commercial or financial relationships that could be construed as a potential conflict of interest.

## Supplementary material

Supplementary Figure S1 Geographical localisation and metallic profiles of sampled lakes. **(a)** Geographical localisation of the sampling sites located in Ryoun-Noranda (West Quebec, Canada) visited in June 2011. Latitute and longitude coordinates of sampling sites are 48.25005489 and −79.40574646 in Opasatica lake (OPA-nc); 48.07601448 and −79.3082428 in Dasserat lake (DAS-lc); 48.24090959 and −79.35012817 in Arnoux Bay (BAR-mc); 48.25051211 and −79.333992 in Arnoux lake (LAR-hc); 48.30474963 and −79.07742262 in Turcotte lake (TUR-hc). This map was produced using Arc GIS Esri® Arc Map™ 10.1 under academic license certification. **(b)** Trace metals concentrations measured in the five sampled lakes 1 year before this study (Laplante and Derome, 2011). The x-axis represents the log ratio of trace metal concentrations (mg/l) and the y-axis represents detection limit in each lake. The metallic gradient showed that Cadmium was under the detection limit in OPA-nc (negative control), at the detection limit in DAS-lc (low contamination), three times more than the detection limit in BAR-mc (medium contamination), LAR-hc (high contamination), and TUR-hc (positive control). Contamination gradient classification refers to the Cadmium log ratio across the five lakes.

Supplementary Figure S2 Classification of lake metacommunities based ORF approach at genus and phylum levels. **(a)** Distribution of ORF and annotated genus in the five metagenomes. This figure showed that not only the number of predicted ORFs (Supplementary File S2), comparable between metagenomes but also the genus count (Supplementary File S3). **(b)** Hierarchical clustering of samples using Ward's method and Bray–Curtis dissimilarity distance, bootstrap AU (Approximately Unbiased) *p*-value and BP (Bootstrap Probability) value are shown on nodes. **(c,d)** principal component analysis (PCA) of samples based on genus relative abundance (RA) assigned with coverage **(c)** and without coverage **(d)** normalization. Metacommunity clustering based on genus abundance is different at phylum level where BAR-mc was closer to OPA-nc and DAS-lc. **(e)** PCA analysis of samples based on function RA with different annotation parameters of alignment length cutoff (30 bp) and identity threshold (60%). **(f)** Distribution of filtered ORFs on different alignment length cutoffs. For the ORF based approach **(a–d)**, the 85% identity threshold, *e*-value of 10^−12^ and minimum alignment length of 50 base pairs parameters were selected in filtering annotations, and the LCA (Lowest Common ancestor) algorithm was used to assign taxonomy.

Supplementary Figure S3 Composition of metacommunities based on the ORFs approach. NMDS (with Bray-Curtis distance) of genera abundance for major abundant phyla fitted to trace metals for *Proteobacteria* **(a)**, *Actinobacteria* **(b)**, *Cyanobacteria* **(c)**, the water pH, and trace metals which correlated significantly with NMDS axes were highlighted in red. Each small point in figures a, b, and c represented the genus abundance, while each big point does represent the lake metacommunities samples using circle shape for OPA-nc in blue and the control TUR-hc in black, and the connected lakes were illustrated with square shape, LAR-hc in red, BAR-mc in orange and DAS-lc in yellow. NMDS loadings (NMDS1, NMDS2), and *P*-value of correlation *r*^2^ of trace metals were reported in Supplementary File S6. Genus plot coordinates, clusters and dot labels are resumed in Supplementary File S4.

Supplementary Figure S4 Hierarchical clustering of taxon and function. **(a)** Hierarchical clustering of artificial replicates based on genus abundance using the subsampled reads approach. **(b)** Hierarchical clustering of samples based on abundance of subsystem level 1 using the ORF approach. **(c, d)** Hierarchical clustering of subsampled replicates based on subsystems level 1 and 3 using the reads approach. Hierarchical clustering was performed using Ward's method and Bray–Curtis dissimilarity distance; bootstrap AU (Approximately Unbiased) *p*-value and BP (Bootstrap Probability) value are shown on the nodes.

Supplementary Figure S5 Heatmap of subsystems in level 1. This heatmap represents metagenomes classification based on subsystems in level 1 (See Supplementary File S5). Dendrogram's topology identified two clusters. The first cluster grouped BAR-mc, LAR-hc and TUR-hc, and the second grouped DAS-lc and OPA-nc. The hierarchical clustering of relative abundance proportions of subsystems, and of samples was performed using Ward's method and Bray–Curtis dissimilarity distance. The ORF approach was used with identity threshold of 85%, *e*-value of 10–12 and minimum alignment length of 50 base pairs parameters. Vegan package and heatmap () function in R were used to produce this figure.

Supplementary Figure S6 Heatmap of subsystems in all levels. Subsystems relative abundance were clustered cross-metagenomes in different levels, level2 (981 modules), level3 (192 modules) and function level (6801 functions) (See Supplementary File S5). The same topology was observed in level 1 (See Supplementary Figure S5) and in all levels. The hierarchical clustering of relative abundance proportions of subsystems, and of samples was performed using Ward's method and Bray–Curtis dissimilarity distance. The ORF approach was used with identity threshold of 60%, *e*-value of 10–12 and minimum alignment length of 50 base pairs parameters. Vegan package and heatmap () function in R were used to produce this figure.

Supplementary Figure S7 Heatmap of multiple subsystems abundant in function level. This heatmap represents cross-metagenomes, the common and most abundant functions (>2%) in 22 subsystems (See Supplementary File S5). Functions of polymetallic resistance (Cation efflux system protein CusA and Cobalt–zinc–cadmium resistance protein CzcA) showed a profile of gradual abundance increase along the pollution gradient. The hierarchical clustering of relative abundance proportions of functions, and of samples was performed using Ward's method and Bray–Curtis dissimilarity distance. The ORF approach was used with identity threshold of 60%, *e*-value of 10–12 and minimum alignment length of 50 base pairs parameters. Vegan package and heatmap () function in R were used to produce this figure.

Supplementary Figure S8 Subsystem of “Phages, prophages, plasmids, and transposable elements” cross-metagenomes. Under this subsystem multiple relevant functions (level 3) related to mobile elements and transfer vectors (Gene transfer agents, transposons, prophages, conjugative plasmids, integrons) were shared between DAS-lc, BAR-mc, LAR-hc, TUR-hc, and depleted in OPA-nc. However, each metagenome contains specific profile of mobile elements functions such like agents of gene transfers and conjugative elements in TUR-hc. The hierarchical clustering of relative abundance proportions of this subsystem modules, and of samples was performed using Ward's method and Bray–Curtis dissimilarity distance. The ORF approach was used with identity threshold of 60%, *e*-value of 10–12 and minimum alignment length of 50 base pairs parameters. Vegan package and heatmap () function in R were used to produce this figure.

Supplementary Figure S9 Metabolic abundance cross-metagenomes. This heatmap represents 1,842 annotated enzymes (See EC number in Supplementary File S7) in all samples. The hierarchical clustering of relative abundance proportions of enzymes, and of samples was performed using Ward's method and Bray–Curtis dissimilarity distance. The dendrogram shows dichotomy between OPA-nc metagenome and all others. The ORF approach was used with identity threshold of 60%, *e*-value of 10–12 and minimum alignment length of 50 base pairs parameters. Vegan package and heatmap () function in R were used to produce this figure.

Supplementary Figure S10 Gradual variation of functions cross-metagenomes. Two heatmaps represent gradual function abundance FP1 (106 functions) and FP2 (123 functions) along the contamination gradient. The hierarchical clustering of relative abundance proportions of functions was performed using Ward's method and Bray–Curtis dissimilarity distance. The ORF approach was used with identity threshold of 60%, *e*-value of 10–12 and minimum alignment length of 50 base pairs parameters. Vegan package and heatmap () function in R were used to produce this figure.

Supplementary Figure S11 Specific variation of functions cross-metagenomes. Two heatmaps represent specific function abundance FP3-OPA-nc (167 functions) and FP3 specific to pollution gradient (225 functions). The hierarchical clustering of relative abundance proportions of functions was performed using Ward's method and Bray–Curtis dissimilarity distance. The ORF approach was used with identity threshold of 60%, *e*-value of 10–12 and minimum alignment length of 50 base pairs parameters. Vegan package and heatmap () function in R were used to produce this figure.

Supplementary File S1 This file contains two tables. The first table resumed the geographical coordinates of sampled sites. The second table presented abiotic parameters measured for each sampled site 1 year before this study (Laplante and Derome, 2011).

Supplementary File S2 This file summarized statistics of reads, contigs, and ORFs MG-RAST annotations per lake metagenome.

Supplementary File S3 This file summarized in one table the relative abundance of major taxa at phylum, class, and genus levels.

Supplementary File S4 This file resumed details of NMDS plots. NMDS loadings, abundance clusters and points labels of all genus (Table 1), *Proteobacteria* (Table 2), *Actinobacteria* (Table 3), *Cyanobacteria* (Table 4), then of all subsystems (Table 5) were reported for assuming a better understanding of NMDS figures and Supplementary Figures.

Supplementary File S5 This file reported subsystems annotations and data analysis; All subsystems data (dataset output of STAMP software) in Table 1, subsystems level 1 relative abundance (RA) in Table 2, list of all annotated subsystems in Table 3, function profiles classification of RA proportions in Table 4, different function profiles (FP) (Tables 5, 6, 7, 8), resume of FP occurrence in subsystems level 1 (Table 9), summary of most abundant functions (Table 10) and summary of the subsystem “*virulence, disease, and defense*” (Table 11).

Supplementary File S6 This file summarized details of NMDS correlation analysis with metadata. Each table resumed NMDS loadings (NMDS1, NMDS2), *P*-value of correlated trace metals of taxa (Table 1), subsystems level 1 (Table 2) and PMRGs.

Supplementary File S7 This file resumed diversity measures based relative abundance of taxa (genus) and function (subsystems) in Table 1 and all annotated enzymes by their EC-number in Table 2.
