# Supplementary figures and images for: Taxon-Function Decoupling as an Adaptive Signature of Lake Microbial Metacommunities Under a Chronic Polymetallic Pollution Gradient
Source: Front Microbiol. 2018 May 3;9:869. doi: 10.3389/fmicb.2018.00869 (PMC5943556; doi:10.3389/fmicb.2018.00869)

# Supplementary figure S1.

a.

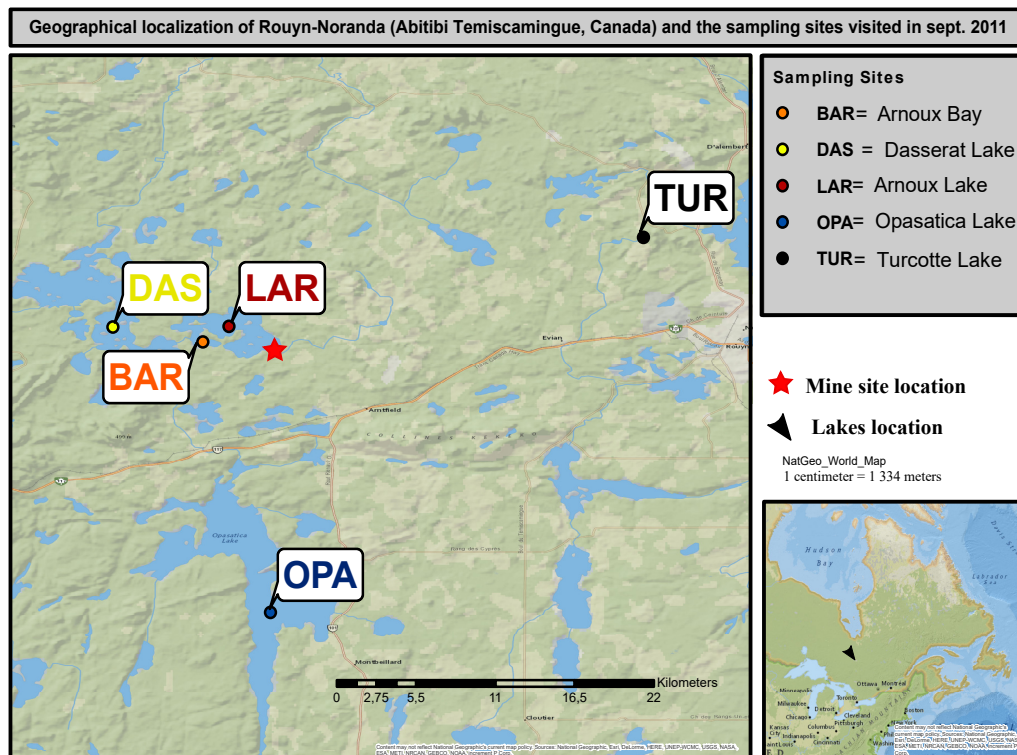

b.

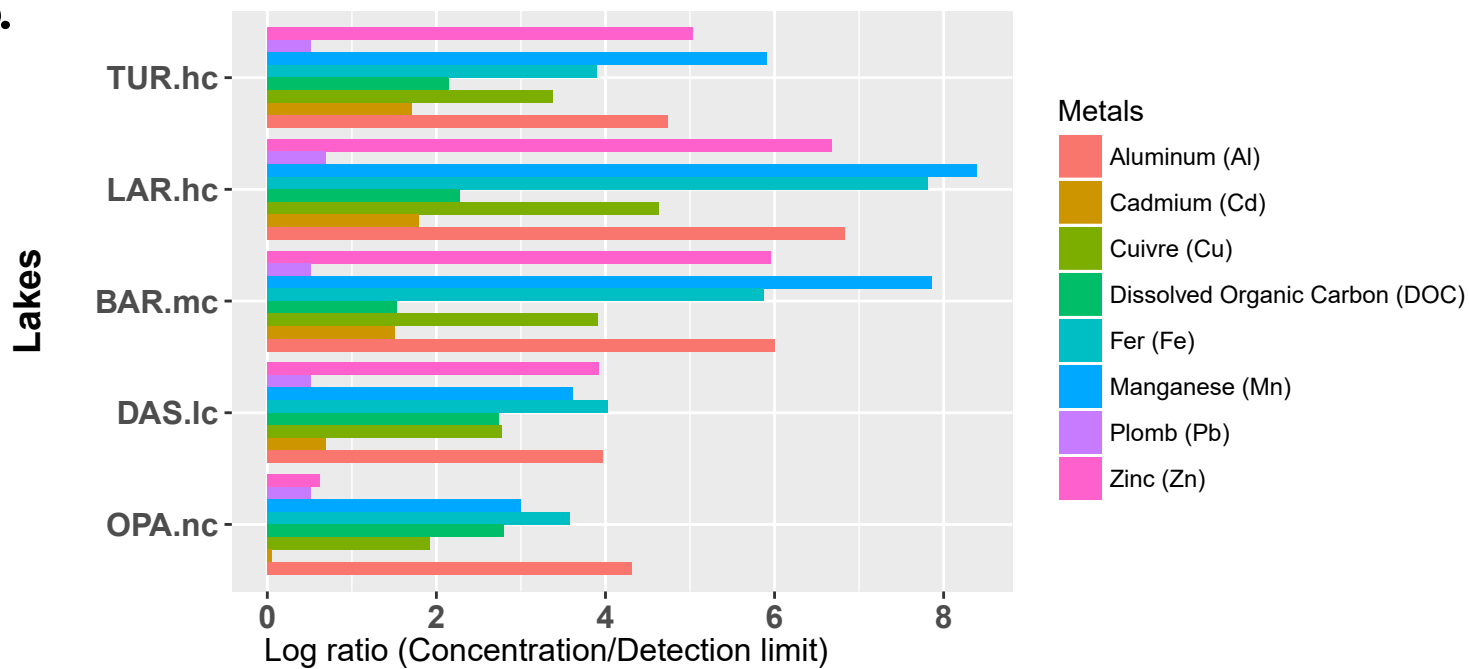

Supplement: Supplementary Figure S1 — Geographical localisation and metallic profiles of sampled lakes. (a) Geographical localisation of the sampling sites located in Ryoun-Noranda (West Quebec, Canada) visited in June 2011. Latitute and longitude coordinates of sampling sites are 48.25005489 and −79.40574646 in Opasatica lake (OPA-nc); 48.07601448 and −79.3082428 in Dasserat lake (DAS-lc); 48.24090959 and −79.35012817 in Arnoux Bay (BAR-mc); 48.25051211 and −79.333992 in Arnoux lake (LAR-hc); 48.30474963 and −79.07742262 in Turcotte lake (TUR-hc). This map was produced using Arc GIS Esri® Arc Map™ 10.1 under academic license certification. (b) Trace metals concentrations measured in the five sampled lakes 1 year before this study (Laplante and Derome, 2011). The x-axis represents the log ratio of trace metal concentrations (mg/l) and the y-axis represents detection limit in each lake. The metallic gradient showed that Cadmium was under the detection limit in OPA-nc (negative control), at the detection limit in DAS-lc (low contamination), three times more than the detection limit in BAR-mc (medium contamination), LAR-hc (high contamination), and TUR-hc (positive control). Contamination gradient classification refers to the Cadmium log ratio across the five lakes. [file Image_1.PDF]

# Supplementary figure S2.

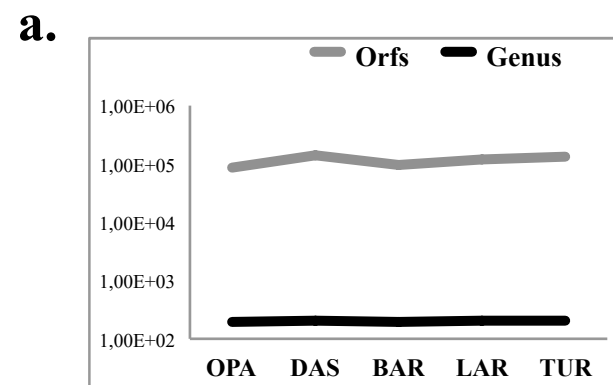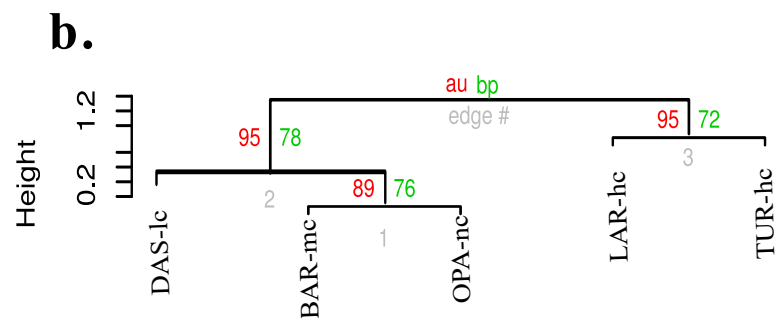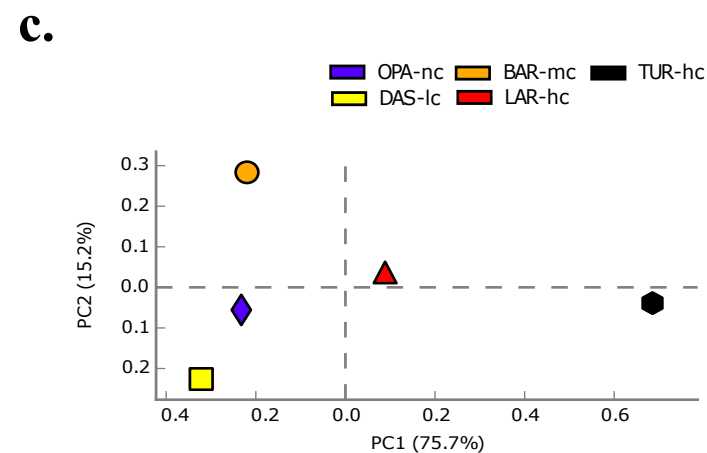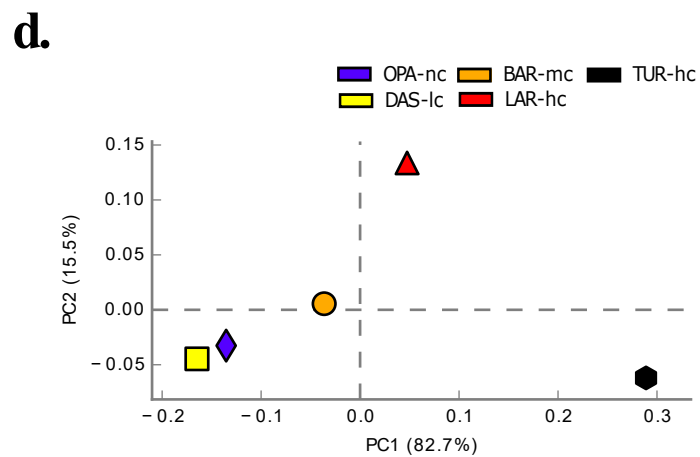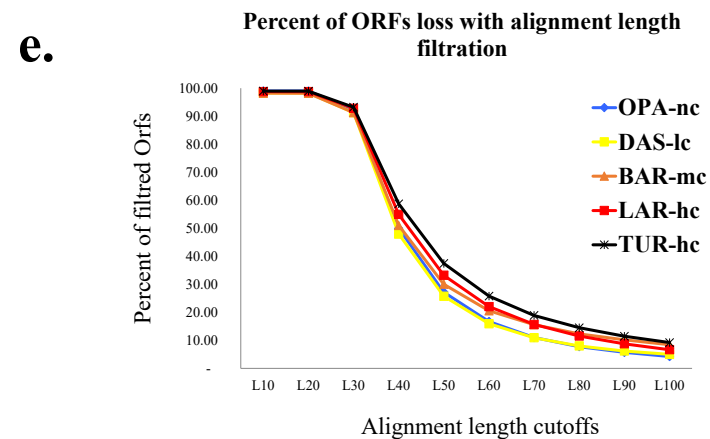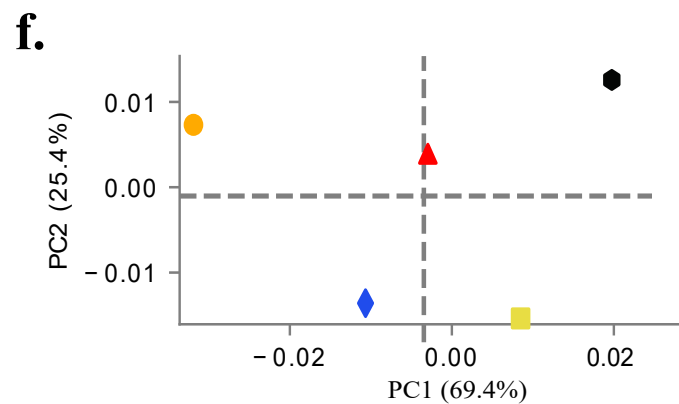

Supplement: Supplementary Figure S2 — Classification of lake metacommunities based ORF approach at genus and phylum levels. (a) Distribution of ORF and annotated genus in the five metagenomes. This figure showed that not only the number of predicted ORFs (Supplementary File S2), comparable between metagenomes but also the genus count (Supplementary File S3). (b) Hierarchical clustering of samples using Ward's method and Bray–Curtis dissimilarity distance, bootstrap AU (Approximately Unbiased) p-value and BP (Bootstrap Probability) value are shown on nodes. (c,d) principal component analysis (PCA) of samples based on genus relative abundance (RA) assigned with coverage (c) and without coverage (d) normalization. Metacommunity clustering based on genus abundance is different at phylum level where BAR-mc was closer to OPA-nc and DAS-lc. (e) PCA analysis of samples based on function RA with different annotation parameters of alignment length cutoff (30 bp) and identity threshold (60%). (f) Distribution of filtered ORFs on different alignment length cutoffs. For the ORF based approach (a–d), the 85% identity threshold, e-value of 10−12 and minimum alignment length of 50 base pairs parameters were selected in filtering annotations, and the LCA (Lowest Common ancestor) algorithm was used to assign taxonomy. [file Image_2.PDF]

# Supplementary figure S4.

a.

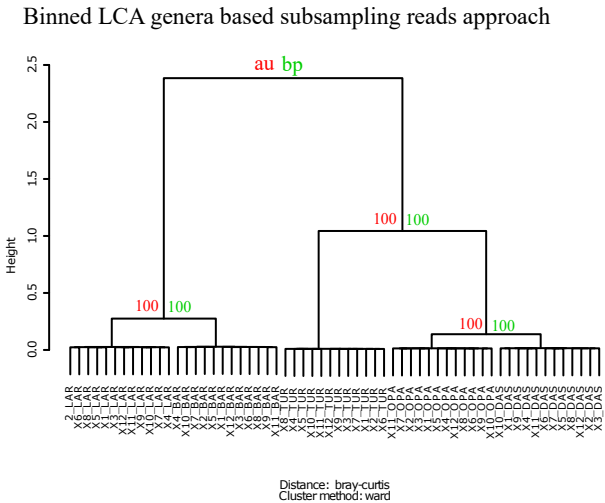

b.

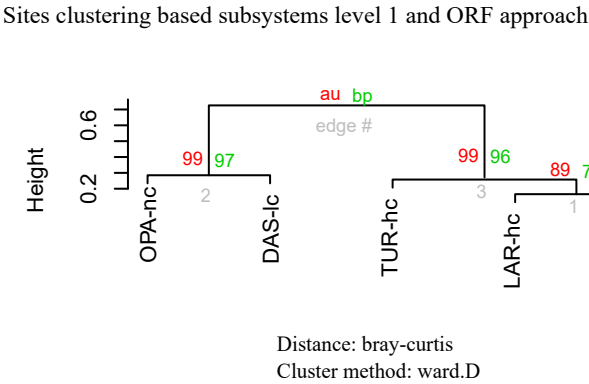

c.

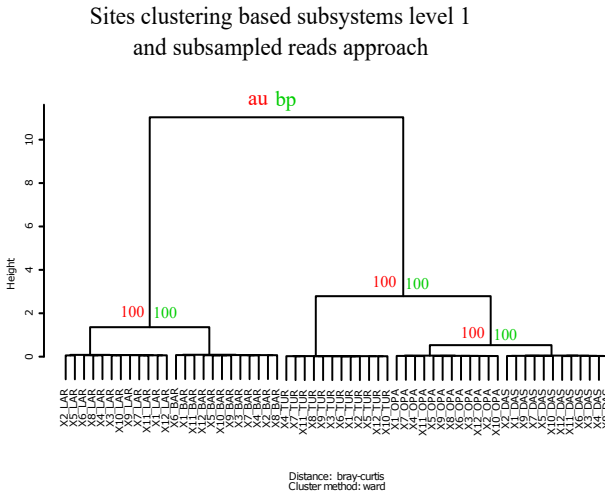

d.

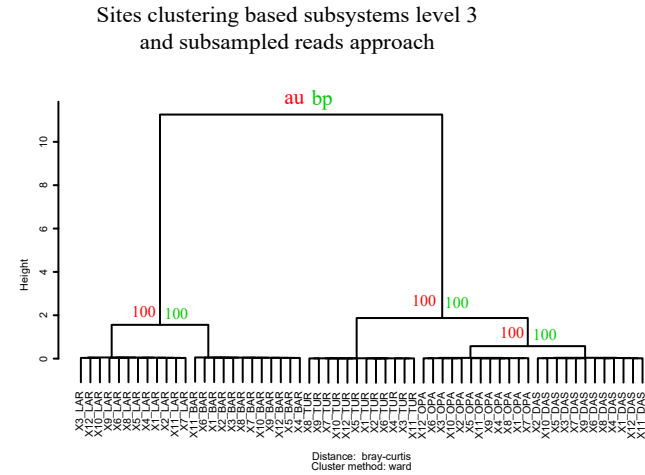

Supplement: Supplementary Figure S4 — Hierarchical clustering of taxon and function. (a) Hierarchical clustering of artificial replicates based on genus abundance using the subsampled reads approach. (b) Hierarchical clustering of samples based on abundance of subsystem level 1 using the ORF approach. (c, d) Hierarchical clustering of subsampled replicates based on subsystems level 1 and 3 using the reads approach. Hierarchical clustering was performed using Ward's method and Bray–Curtis dissimilarity distance; bootstrap AU (Approximately Unbiased) p-value and BP (Bootstrap Probability) value are shown on the nodes. [file Image_4.PDF]

# Supplementary figure S5.

Heatmap of susbsytems in level 1 cross-metagenomes

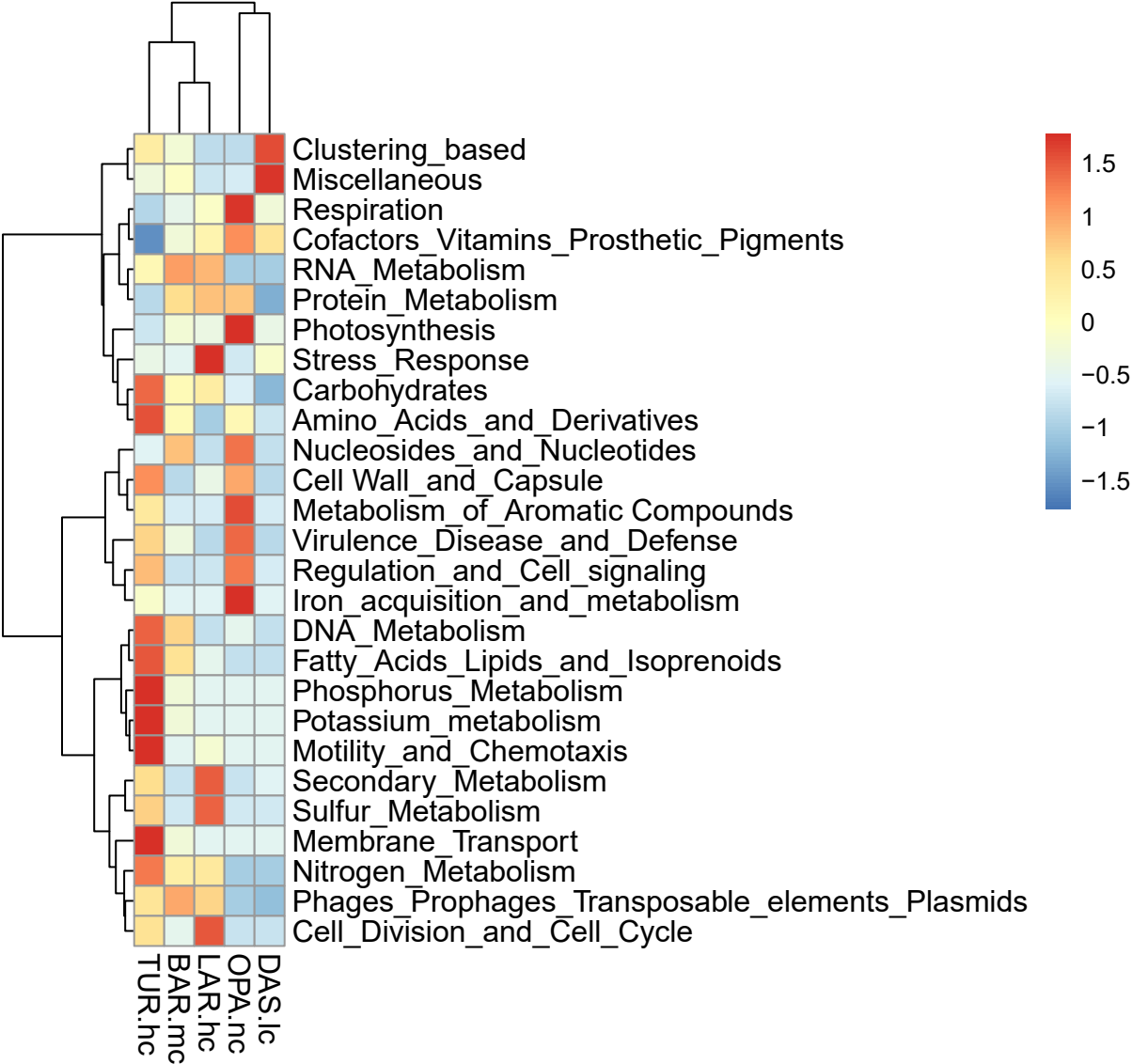

Supplement: Supplementary Figure S5 — Heatmap of subsystems in level 1. This heatmap represents metagenomes classification based on subsystems in level 1 (See Supplementary File S5). Dendrogram's topology identified two clusters. The first cluster grouped BAR-mc, LAR-hc and TUR-hc, and the second grouped DAS-lc and OPA-nc. The hierarchical clustering of relative abundance proportions of subsystems, and of samples was performed using Ward's method and Bray–Curtis dissimilarity distance. The ORF approach was used with identity threshold of 85%, e-value of 10–12 and minimum alignment length of 50 base pairs parameters. Vegan package and heatmap () function in R were used to produce this figure. [file Image_5.PDF]

Supplementary figure 6.

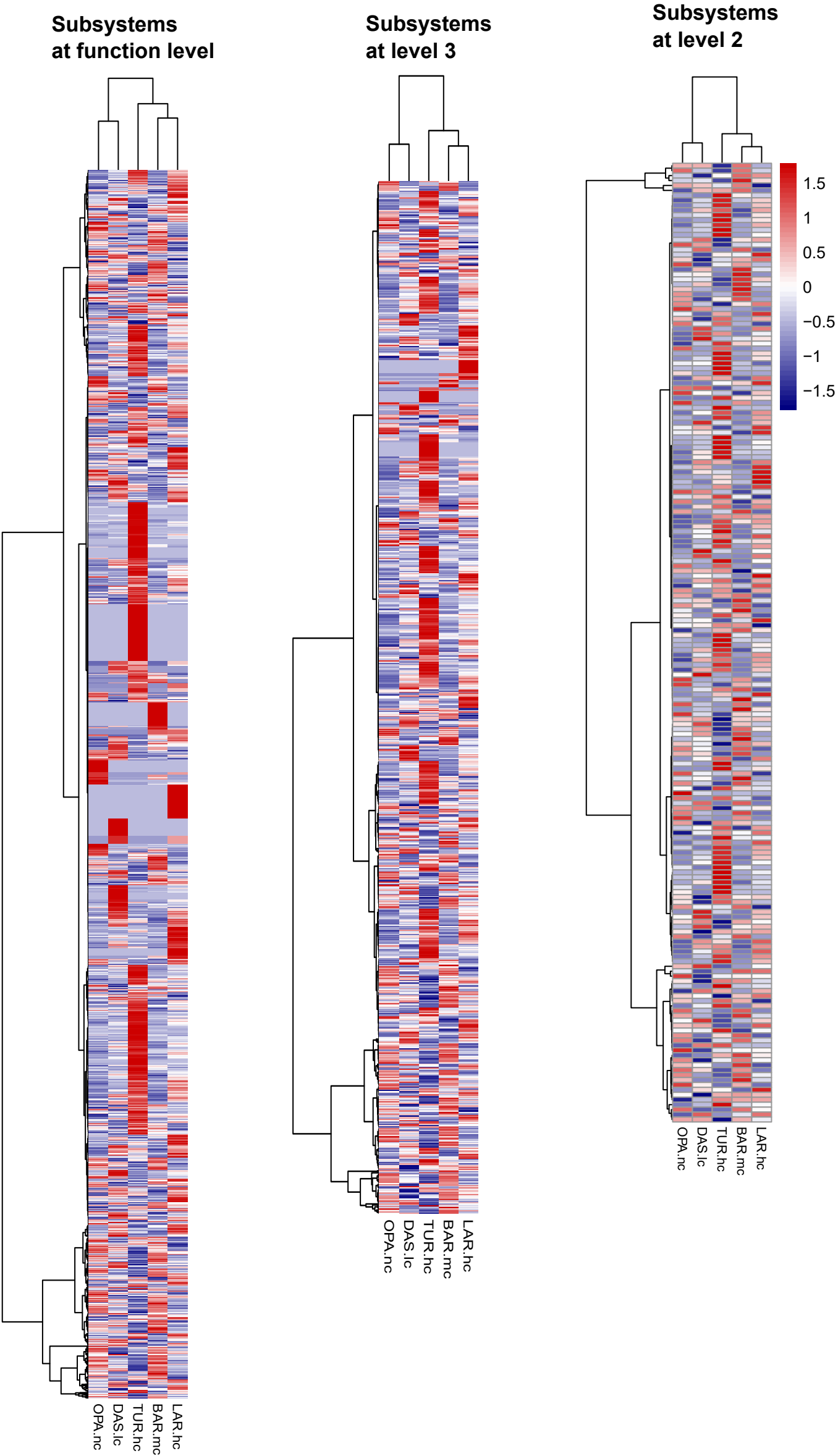

Supplement: Supplementary Figure S6 — Heatmap of subsystems in all levels. Subsystems relative abundance were clustered cross-metagenomes in different levels, level2 (981 modules), level3 (192 modules) and function level (6801 functions) (See Supplementary File S5). The same topology was observed in level 1 (See Supplementary Figure S5) and in all levels. The hierarchical clustering of relative abundance proportions of subsystems, and of samples was performed using Ward's method and Bray–Curtis dissimilarity distance. The ORF approach was used with identity threshold of 60%, e-value of 10–12 and minimum alignment length of 50 base pairs parameters. Vegan package and heatmap () function in R were used to produce this figure. [file Image_6.PDF]

Supplementary figure S7.

Profiles of most abundant functions from multiple subsystems

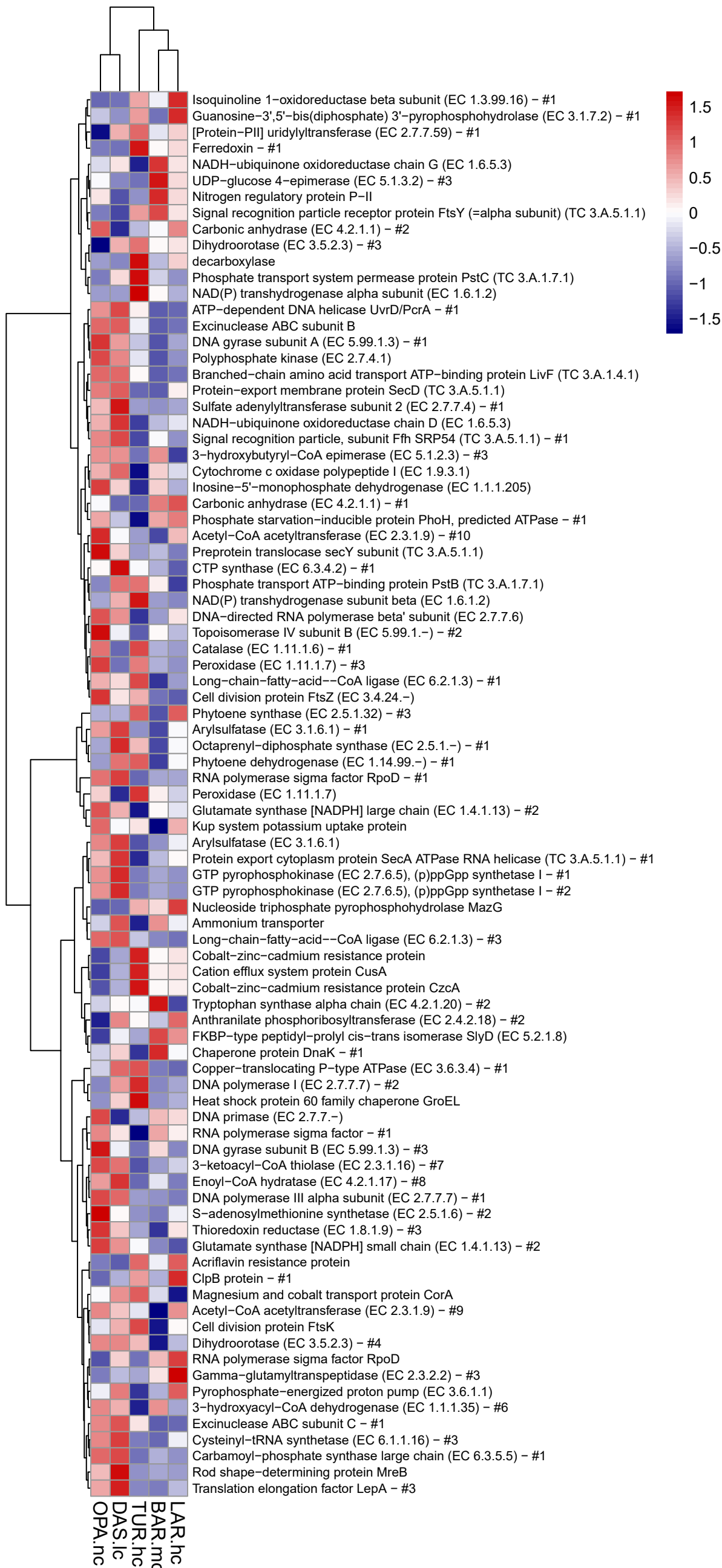

Supplement: Supplementary Figure S7 — Heatmap of multiple subsystems abundant in function level. This heatmap represents cross-metagenomes, the common and most abundant functions (>2%) in 22 subsystems (See Supplementary File S5). Functions of polymetallic resistance (Cation efflux system protein CusA and Cobalt–zinc–cadmium resistance protein CzcA) showed a profile of gradual abundance increase along the pollution gradient. The hierarchical clustering of relative abundance proportions of functions, and of samples was performed using Ward's method and Bray–Curtis dissimilarity distance. The ORF approach was used with identity threshold of 60%, e-value of 10–12 and minimum alignment length of 50 base pairs parameters. Vegan package and heatmap () function in R were used to produce this figure. [file Image_7.PDF]

# Supplementary Figure 8.

Phages, Prophages, Transposable elements, Plasmids subsystem at level 3

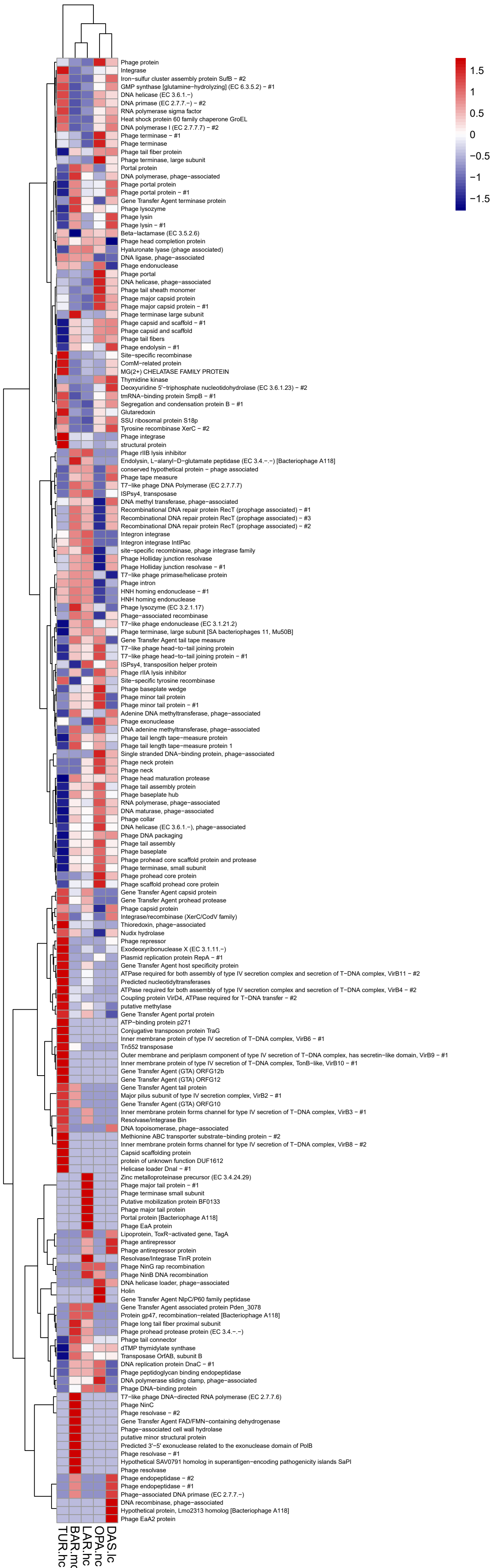

Supplement: Supplementary Figure S8 — Subsystem of “Phages, prophages, plasmids, and transposable elements” cross-metagenomes. Under this subsystem multiple relevant functions (level 3) related to mobile elements and transfer vectors (Gene transfer agents, transposons, prophages, conjugative plasmids, integrons) were shared between DAS-lc, BAR-mc, LAR-hc, TUR-hc, and depleted in OPA-nc. However, each metagenome contains specific profile of mobile elements functions such like agents of gene transfers and conjugative elements in TUR-hc. The hierarchical clustering of relative abundance proportions of this subsystem modules, and of samples was performed using Ward's method and Bray–Curtis dissimilarity distance. The ORF approach was used with identity threshold of 60%, e-value of 10–12 and minimum alignment length of 50 base pairs parameters. Vegan package and heatmap () function in R were used to produce this figure. [file Image_8.PDF]

# Supplementary Figure 9.

Profiles of metabolites (EC number) abundance

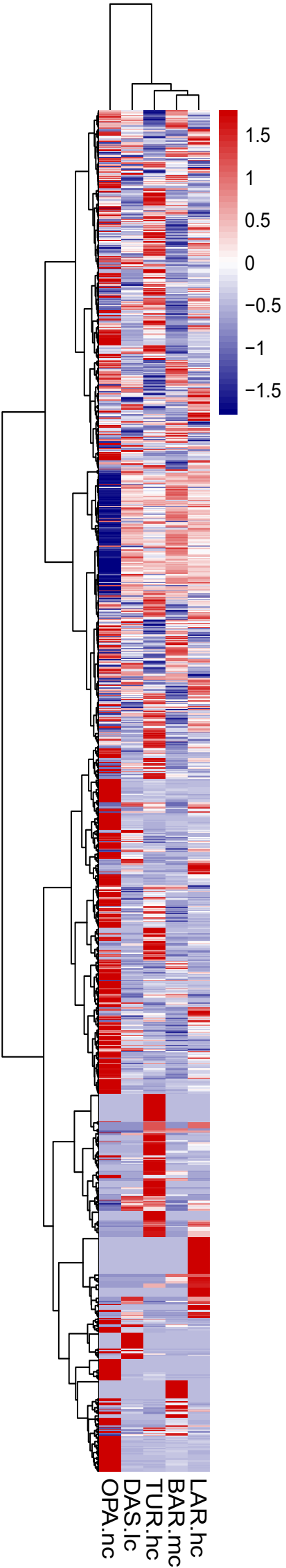

Supplement: Supplementary Figure S9 — Metabolic abundance cross-metagenomes. This heatmap represents 1,842 annotated enzymes (See EC number in Supplementary File S7) in all samples. The hierarchical clustering of relative abundance proportions of enzymes, and of samples was performed using Ward's method and Bray–Curtis dissimilarity distance. The dendrogram shows dichotomy between OPA-nc metagenome and all others. The ORF approach was used with identity threshold of 60%, e-value of 10–12 and minimum alignment length of 50 base pairs parameters. Vegan package and heatmap () function in R were used to produce this figure. [file Image_9.PDF]
