# Supplementary material for: Taxon-Function Decoupling as an Adaptive Signature of Lake Microbial Metacommunities Under a Chronic Polymetallic Pollution Gradient
Source: Front Microbiol. 2018 May 3;9:869. doi: 10.3389/fmicb.2018.00869 (PMC5943556; doi:10.3389/fmicb.2018.00869)

Supplementary figure S3.

a.

NMDS of Proteobacteria genus abundance fitted to trace metals

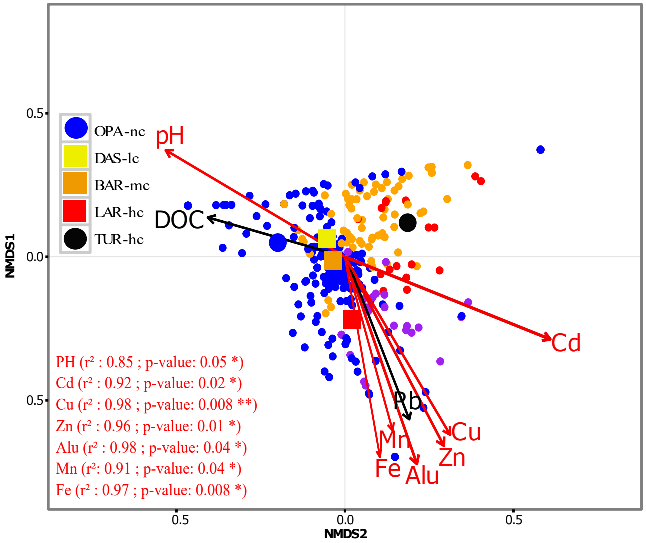

b.

NMDS of Actinobacteria genus abundance fitted to trace metals

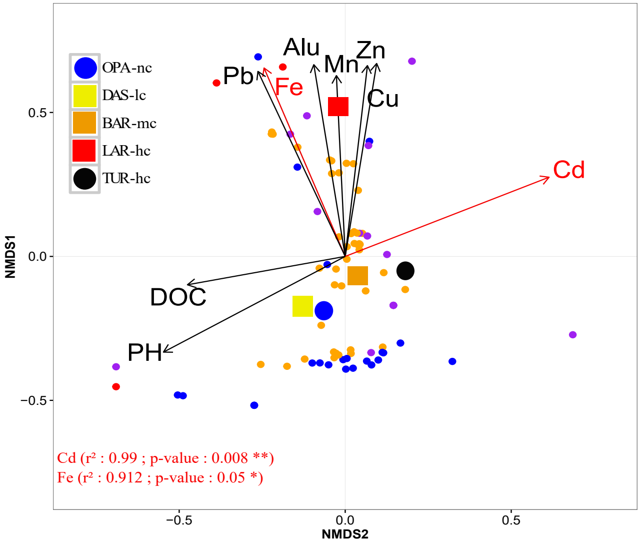

c.

NMDS of Cyanobacteria genus abundance fitted to trace metals

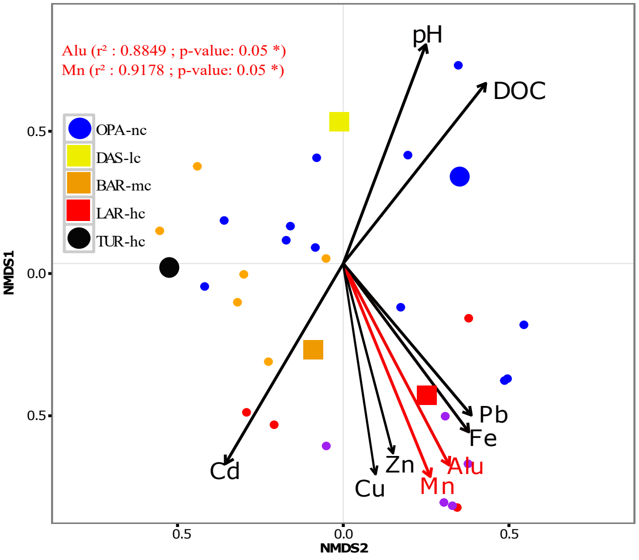

Supplement: Supplementary Figure S3 — Composition of metacommunities based on the ORFs approach. NMDS (with Bray-Curtis distance) of genera abundance for major abundant phyla fitted to trace metals for Proteobacteria (a), Actinobacteria (b), Cyanobacteria (c), the water pH, and trace metals which correlated significantly with NMDS axes were highlighted in red. Each small point in figures a, b, and c represented the genus abundance, while each big point does represent the lake metacommunities samples using circle shape for OPA-nc in blue and the control TUR-hc in black, and the connected lakes were illustrated with square shape, LAR-hc in red, BAR-mc in orange and DAS-lc in yellow. NMDS loadings (NMDS1, NMDS2), and P-value of correlation r2 of trace metals were reported in Supplementary File S6. Genus plot coordinates, clusters and dot labels are resumed in Supplementary File S4. [file Image_3.PDF]
