# Supplementary material for: Taxon-Function Decoupling as an Adaptive Signature of Lake Microbial Metacommunities Under a Chronic Polymetallic Pollution Gradient
Source: Front Microbiol. 2018 May 3;9:869. doi: 10.3389/fmicb.2018.00869 (PMC5943556; doi:10.3389/fmicb.2018.00869)

Supplementary figure S10.

A. Function gradual increase (FP2) along the pollution gradient

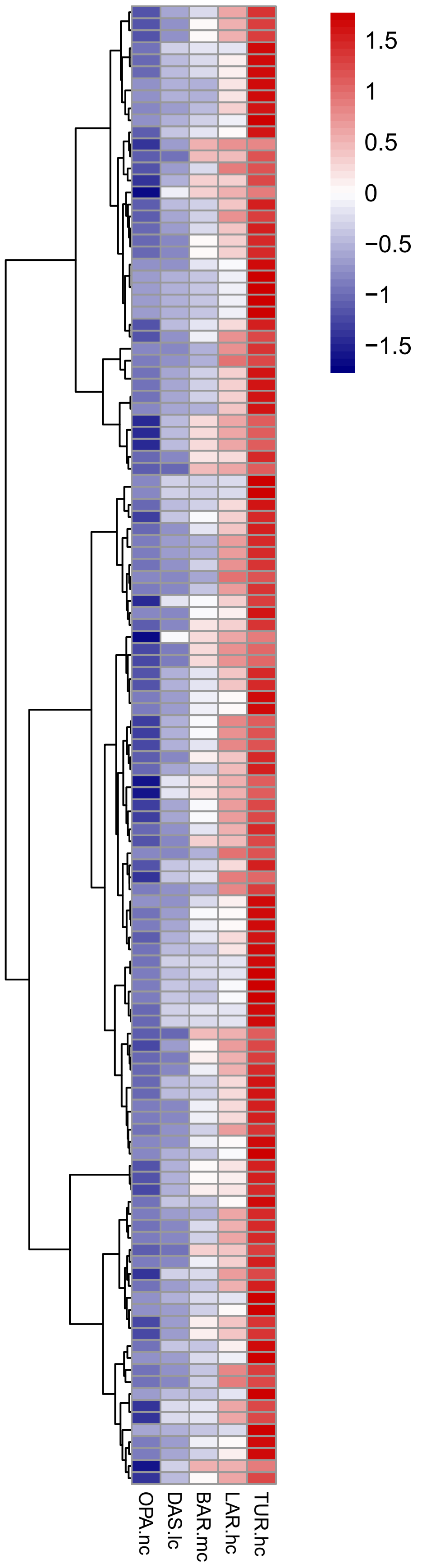

B. Function gradual decrease (FP1) along the pollution gradient

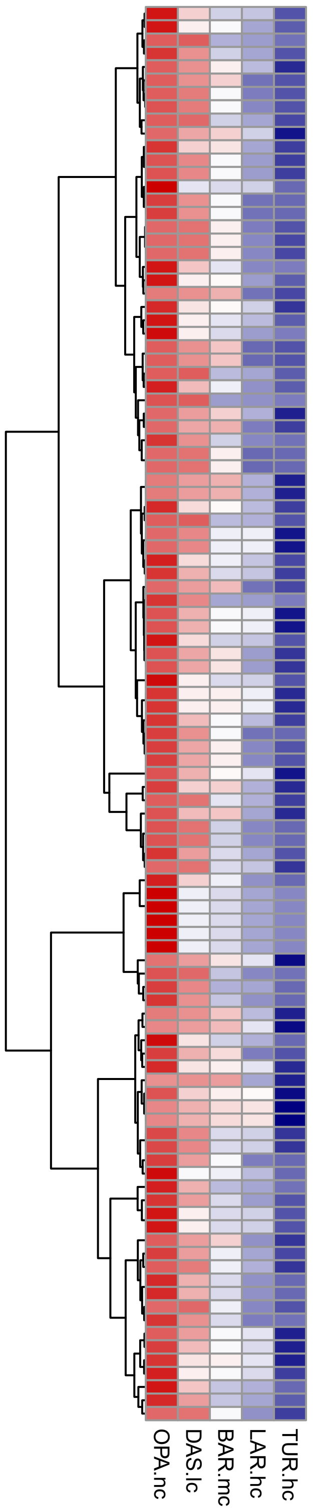

Supplement: Supplementary Figure S10 — Gradual variation of functions cross-metagenomes. Two heatmaps represent gradual function abundance FP1 (106 functions) and FP2 (123 functions) along the contamination gradient. The hierarchical clustering of relative abundance proportions of functions was performed using Ward's method and Bray–Curtis dissimilarity distance. The ORF approach was used with identity threshold of 60%, e-value of 10–12 and minimum alignment length of 50 base pairs parameters. Vegan package and heatmap () function in R were used to produce this figure. [file Image_10.PDF]
