# Supplementary material for: Taxon-Function Decoupling as an Adaptive Signature of Lake Microbial Metacommunities Under a Chronic Polymetallic Pollution Gradient
Source: Front Microbiol. 2018 May 3;9:869. doi: 10.3389/fmicb.2018.00869 (PMC5943556; doi:10.3389/fmicb.2018.00869)

Supplementary figure S11.

A. Specific functions in OPA-nc

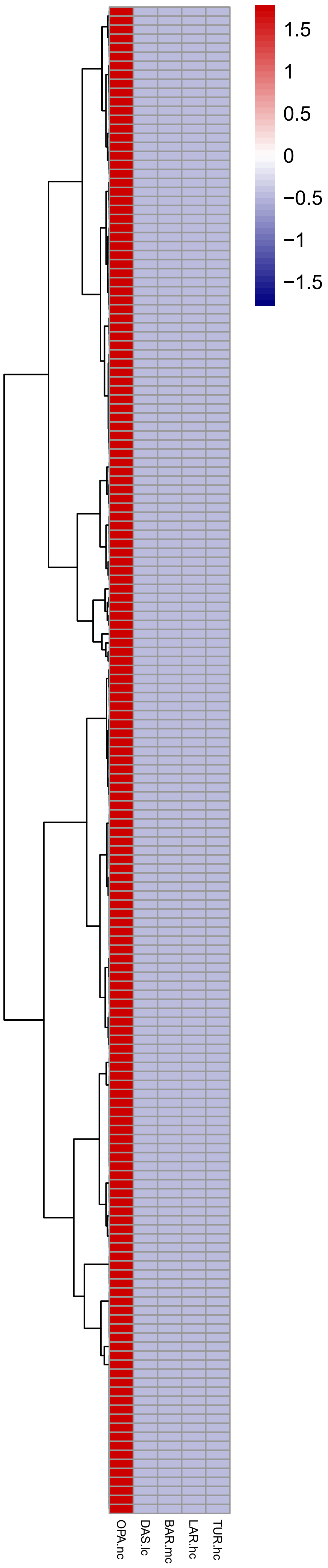

B. Specific functions along the pollution gradient

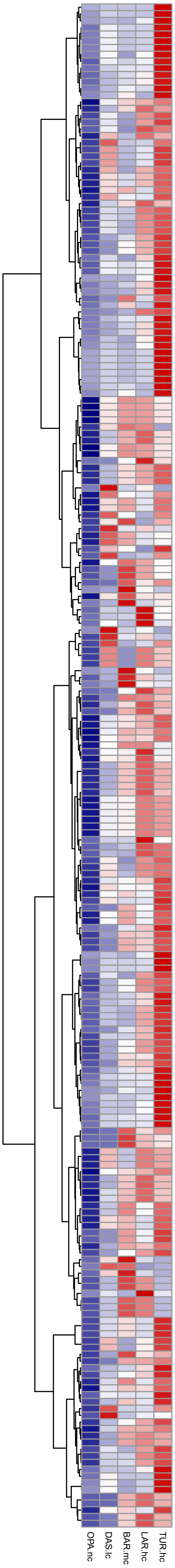

Supplement: Supplementary Figure S11 — Specific variation of functions cross-metagenomes. Two heatmaps represent specific function abundance FP3-OPA-nc (167 functions) and FP3 specific to pollution gradient (225 functions). The hierarchical clustering of relative abundance proportions of functions was performed using Ward's method and Bray–Curtis dissimilarity distance. The ORF approach was used with identity threshold of 60%, e-value of 10–12 and minimum alignment length of 50 base pairs parameters. Vegan package and heatmap () function in R were used to produce this figure. [file Image_11.PDF]
